# A novel kinetic model to demonstrate the independent effects of ATP and ADP/Pi concentrations on sarcomere function

**DOI:** 10.1371/journal.pcbi.1012321

**Published:** 2024-08-05

**Authors:** Andrew A. Schmidt, Alexander Y. Grosberg, Anna Grosberg

**Affiliations:** 1 Department of Biomedical Engineering, University of California, Irvine, Irvine, California, United States of America; 2 UCI Edwards Lifesciences Foundation Cardiovascular Innovation and Research Center (CIRC), University of California, Irvine, Irvine, California, United States of America; 3 Department of Physics and Center for Soft Matter Research, New York University, New York, New York, United States of America; 4 Department of Chemical & Biomolecular Engineering, University of California, Irvine, Irvine, California, United States of America; 5 The NSF-Simons Center for Multiscale Cell Fate Research and Sue and Bill Gross Stem Cell Research Center and Center for Complex Biological Systems, University of California, Irvine, Irvine, California, United States of America; University of Michigan, UNITED STATES OF AMERICA

## Abstract

Understanding muscle contraction mechanisms is a standing challenge, and one of the approaches has been to create models of the sarcomere–the basic contractile unit of striated muscle. While these models have been successful in elucidating many aspects of muscle contraction, they fall short in explaining the energetics of functional phenomena, such as rigor, and in particular, their dependence on the concentrations of the biomolecules involved in the cross-bridge cycle. Our hypothesis posits that the stochastic time delay between ATP adsorption and ADP/Pi release in the cross-bridge cycle necessitates a modeling approach where the rates of these two reaction steps are controlled by two independent parts of the total free energy change of the hydrolysis reaction. To test this hypothesis, we built a two-filament, stochastic-mechanical half-sarcomere model that separates the energetic roles of ATP and ADP/Pi in the cross-bridge cycle’s free energy landscape. Our results clearly demonstrate that there is a nontrivial dependence of the cross-bridge cycle’s kinetics on the independent concentrations of ATP, ADP, and Pi. The simplicity of the proposed model allows for analytical solutions of the more basic systems, which provide novel insight into the dominant mechanisms driving some of the experimentally observed contractile phenomena.

## Author summary

Explaining the intricate workings behind muscle contraction remains a fundamental challenge in our field. In this work, we develop a computational model of the sarcomere designed to unravel the basic energetics of sarcomere contraction, and we place major emphasis on the stochastic nature of the reactions in the cross-bridge cycle. The main goal was to illustrate how dynamic processes such as rigor are contingent upon the concentrations of biomolecules governing the kinetics of the cross-bridge cycle. We posited that including the free energy contributions associated with ATP and ADP/Pi as separate reaction steps could unveil previously inaccessible aspects of sarcomere contraction. To test this hypothesis, we constructed a stochastic-mechanical half-sarcomere model whose kinetics explicitly account for the fact that ATP and ADP/Pi interact with myosin at different times in the cross-bridge cycle. Our findings demonstrate a dependence of sarcomere outputs on independent concentrations of ATP, ADP, and Pi, a phenomenon exclusively reproducible with our hypothesized free energy framework. Lastly, the conceptual simplicity of our model enables analytical solutions for elementary systems, affording new insights into the principal drivers governing experimentally observed contractile phenomena.

## Introduction

Force generation in striated muscle is regulated by the complex interactions between the actomyosin complex and ATP, ADP, and inorganic phosphate (Pi). Changes in these concentrations can significantly affect muscle contraction, relaxation, and the overall energy balance of contractile cells. Both decreased ATP levels and elevated ADP and Pi levels have been observed in several pathological conditions including heart failure, ischemia, and mitochondrial disorders [1–5]. Despite the importance of investigating the effects of varying ATP, ADP, and Pi concentrations on muscle, the mechanisms that drive the dynamical contractile response are not fully understood.

The generation and maintenance of contractile mechanical stress in striated muscle is performed by sarcomeres, the basic contractile units of striated muscle. Sarcomeres consist of a three-dimensional lattice of two main types of filaments–thick filaments, which are bound to the center of the sarcomere at the M-line and the ends of the sarcomere at the Z-lines (via titin), and thin filaments, which are bound only at the Z-lines [6, 7]. During concentric contraction, the sarcomere shortens as thick filament myosin heads pull thin filaments toward the center of the sarcomere. This pulling force is generated via the cross-bridge cycle, which involves interactions between a single myosin head and a discrete binding site on the actin filament [7, 8]. In each cycle, myosin ATPase hydrolyzes one ATP molecule, whose free energy of hydrolysis is partially converted into mechanical work during the power stroke [8, 9]. Consequently, ATP availability and the ease of release of its hydrolysis products, ADP and Pi, play integral roles in the possible force generated by the muscle. Therefore, recreating these dynamics of the cross-bridge cycle could be essential for sarcomere models.

For over half a century, a variety of models have been developed to recapitulate the behavior of a sarcomere [10–27]. A review of these models can be found within Niederer et al.’s work [28] and within the Introduction of Mijailovich et al.’s 2016 MUSICO paper [21]. Many of the stochastic, spatially explicit models recreate the discrete locations and interactions of the sarcomeric filaments in space (1 to 3 dimensions), allow them to capture the nuances of force generation at a granular level, the propagation of mechanical signals, the heterogeneity in cross-bridge binding and sarcomere lengths, and the internal tension contributions by other compliant components of the sarcomere. However, the kinetic schema of some of these existing models needs to be augmented with rate constants that properly include the free energy contributions of the concentrations of ATP, ADP, and Pi in order to cover a wider variety of experimental conditions [14–23]. A physiologically relevant partitioning of these chemical potentials would also allow for closer examination of the effects of ATP availability, as well as ADP and Pi excess, on sarcomere force generation and maintenance. Conversely, while some probabilistic sarcomere models include the impact of all three molecules in their cross-bridge rate kinetics [25–27], they do not possess the same advantages as stochastic and discrete lattice sarcomere models [29, 30].

In this work, we detail the formulation of a spatially explicit, two-filament half-sarcomere model capable of elucidating force generation profiles at varying levels of ATP, ADP, and Pi. Specifically, we employed this model to predict the sarcomeric ATP consumption associated with different levels of contractile force. Thus, we created a novel stochastic-mechanical sarcomere model that tracks discrete node locations and implements a direct dependence of cross-bridge rate kinetics on the concentrations of ATP and its hydrolysis products. The findings of this work yield new insight on the energetics of force generation in muscle tissues.

## Methods: Model formulation

We adopt, with small modifications, the two-filament sarcomere model analyzed in previous studies [10, 14, 16]. Our model is a composition of three aspects, the first of which is the half-sarcomere’s geometry. This aspect simplifies the three-dimensional interactions between thick and thin filaments to a one-dimensional system. The second aspect, the mechanics of the half-sarcomere, assumes the sarcomere behaves as a set of linearly elastic (Hookean) springs, and is described by a set of linear equations that combine the geometric constants of the sarcomere with the spring constants of the sarcomere’s physiological components. The final aspect of this model, which underpins the innovation introduced in this paper, is the chemical kinetics, which describe the stochastic chemical transformations through the cross-bridge cycle. Following the majority of previous works, we assume that the elastic equilibrium in the system is achieved comparatively very fast, such that chemical transformations occur essentially between various elastically equilibrated states. To describe the chemical cycle, we adopt the middle ground between the simplest two-state models [10] and 9 state models [30], and employ the three-state description, which is widely considered the minimal number of states appropriate for recapitulating a cross-bridge’s biomechanics [10, 14, 16, 21].

### Geometry

**Fig 1:**
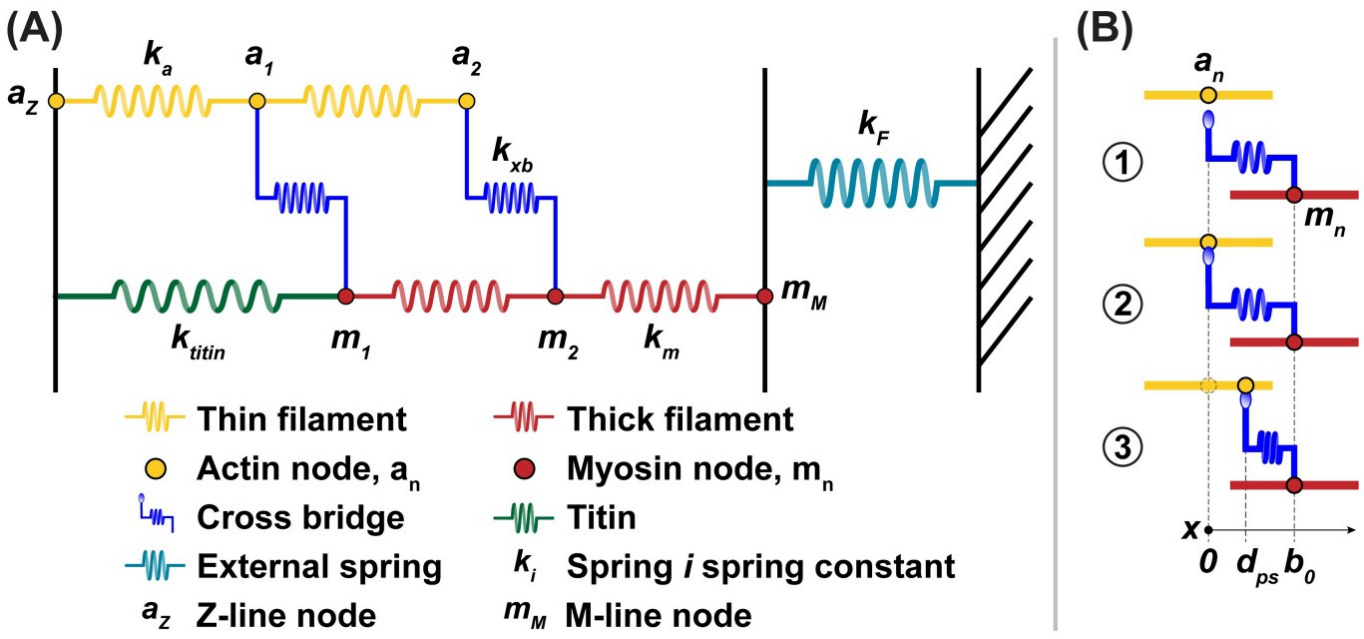
Schematic of a half-sarcomere. (A) Half-sarcomere model showing the geometry of a system containing two actin nodes and two myosin nodes. Cross-bridges are bound, connecting the thick and thin filaments. (B) Schematic showing the three potential mechanical states of a cross-bridge. State 1 shows a cross-bridge unbound from the thin filament. State 2 shows a bound, pre-power stroke cross-bridge in a low force bearing state. State 3 shows a bound, post-power stroke cross-bridge in a high force bearing state. In state 3, the cross-bridge has also undergone a conformational change where the cross-bridge rest length (*b*_0_) has shortened by the length of a power stroke (*d*_*ps*_). The model is one dimensional, but this figure illustrates the model in two dimensions for clarity. All forces in this model are assumed to be one-dimensional, parallel to the filaments.

The geometry of the half-sarcomere includes two filaments that are each composed of an array of nodes (Fig 1). Each node on a thin filament (*a*_*n*_) represents a discrete actin binding site to which a myosin cross-bridge can bind. Thick filament nodes (*m*_*n*_) represent the base of each myosin cross-bridge. In addition to the total number of actin nodes, *N*_*a*_, and the total number myosin nodes, *N*_*m*_, there is a node at the end of each filament: one at the Z-line (*a*_*Z*_) and the M-line (*m*_*M*_) for the thin and thick filaments respectively. This results in *N*_*a*_ + *N*_*m*_ + 2 total nodes. Titin was incorporated into this model as a spring element binding the Z-line to the myosin node most distal to the M-line (Fig 1A, green spring). While Fig 1 is depicted in two dimensions, it is only done so for the clarity of presentation. The forces and displacements in the model are assumed to exist solely along the x-axis parallel to the thick and thin filaments. Elements of physiological spacing were incorporated into this study’s model in order to preserve, at least in part, the properties of the higher order three-dimensional nature of physiological sarcomeres (details in Section A of S1 Text). While this one-dimensional, two-filament system does not fully capture the three-dimensional helical geometry of a sarcomere *in vivo*, the simplicity of the system makes it a valuable tool for interrogating the mechanical and chemical dynamics of force generation relevant to this paper.

### Mechanics

The nodes of the sarcomere model are connected by Hookean springs. All springs have elastic spring constants corresponding to the physiological component they represent (Fig 1A). The cross-bridge springs can be in different positions (Fig 1B) and will exert different forces depending on the myosin state. The mechanical force balances at each node were formulated in terms of a system of linear equations. The actin and myosin node locations (with the exception of the Z-line node fixed at position *x* = 0) were represented in a single vector ***P***. A matrix of the relevant spring constants were stored in ***K***, and lastly, boundary conditions and filament rest lengths were represented in vector ***V***:

$$\begin{array}{c} P=K^{-1}V \end{array}$$ (1)

As cross-bridge binding and/or force generation within the half-sarcomere causes distortions in the spring elements of the model, both ***K*** and ***V*** vary their components accordingly. At any moment, the mechanical equilibrium of the model, and more specifically the location of each node within the lattice, was calculated from Eq 1 using MATLAB’s internal system of linear equations solver (details in Section B of S1 Text).

### Kinetics

**Fig 2:**
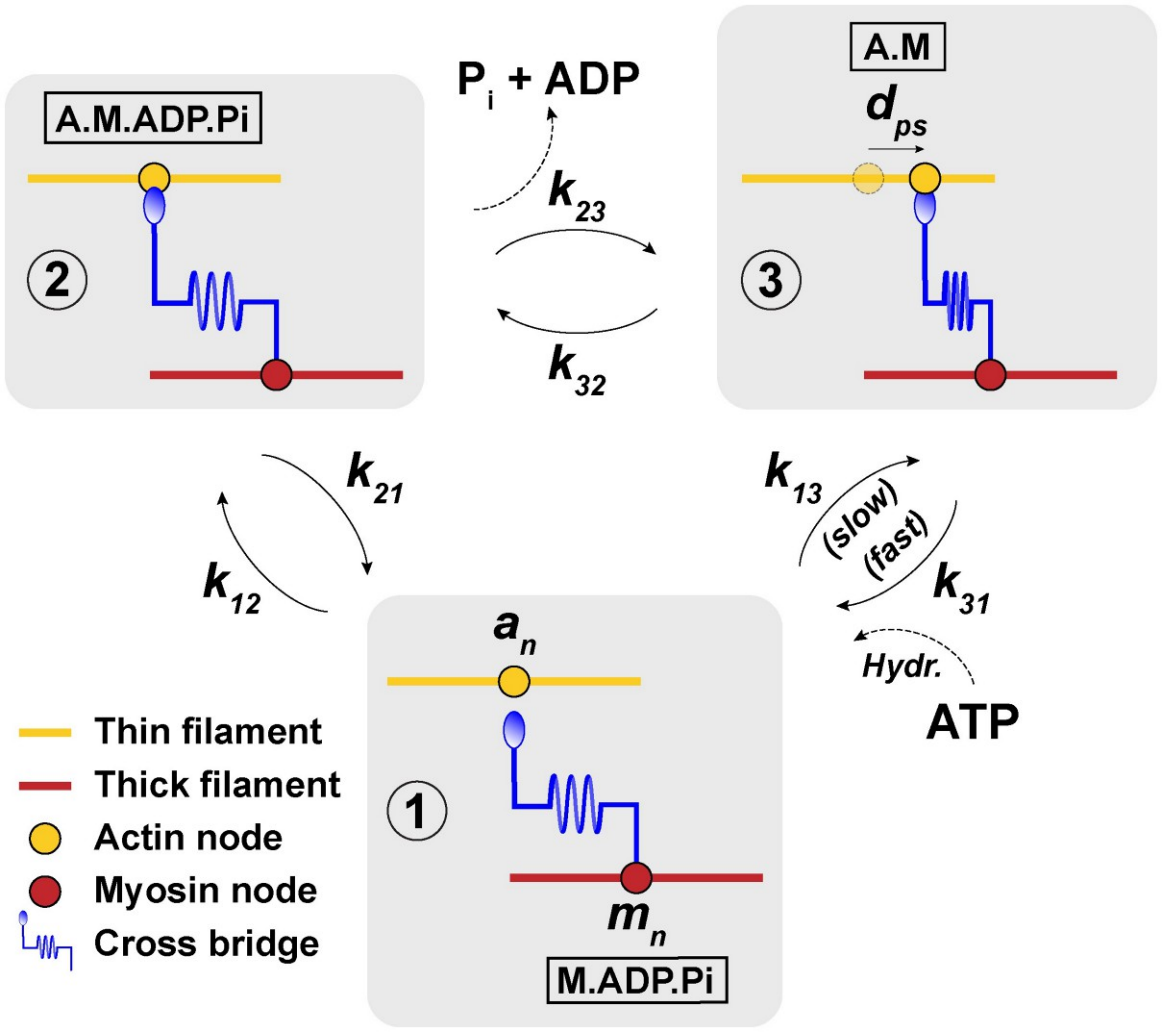
Schematic of the three-state system. Shorthand for the biochemical states of the actomyosin complex are displayed in black frames: A-actin, M-myosin, ATP-adenosine triphophate, ADP-adenosine diphosphate, Pi-inorganic phosphate. All elements in each black box are bound. Between state 3 and state 1, ATP binds the actomyosin complex and is hydrolyzed. Rates for the transitions between each state are labeled such that *k*_*ij*_ represents the transition rate from state *i* to *j*. Association of ATP to the actomyosin complex and the dissociation of ADP and Pi from the actomyosin complex are indicated at the appropriate transition.

**Fig 3:**
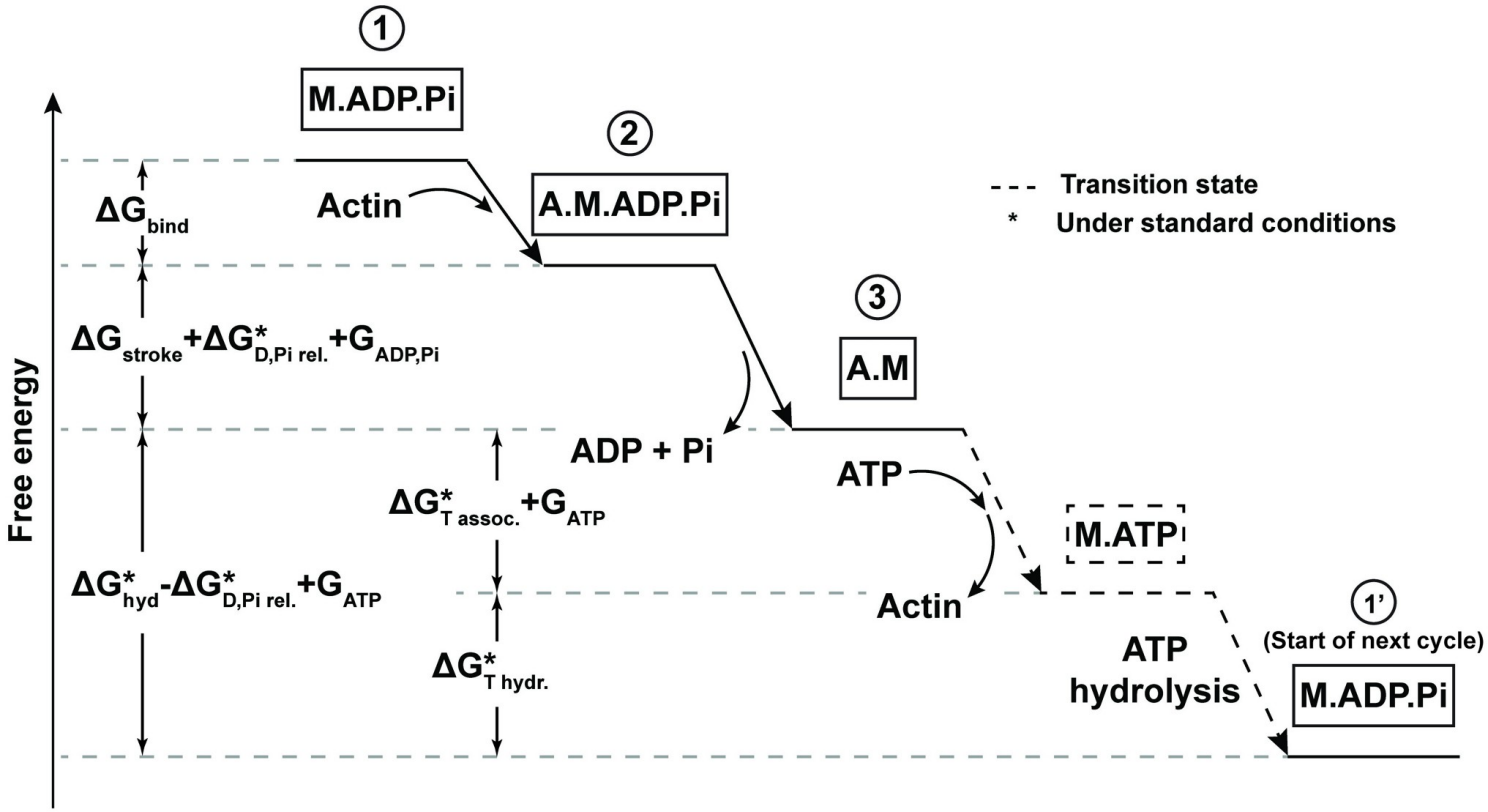
Schematic of free energy landscape for one full cycle. Shorthand of the biochemical states of the actomyosin complex are framed in black (key in Fig 2 caption). Transition states are denoted by dashed lines and dashed frames. Δ*G*_hyd_ = Δ*G*_T assoc._ + Δ*G*_T hydr._ + Δ*G*_D,Pi rel._. Free energy of association of ATP is Δ*G*_*T assoc*._. Free energy of ATP hydrolysis is Δ*G*_T hydr._. Free energy of of ADP and Pi release from actomyosin is Δ*G*_D,Pi rel._. *G*_ADP,Pi_ = *k*_B_*T* ln([ADP][Pi]). *G*_ATP_ = *k*_B_*T* ln([ATP]). Note: The free energies of each state depicted in this free energy landscape assume there is no cross-bridge deformation, and therefore do not include the elastic potential energy contributions of such deformations. The complete free energies of each state, including elastic potential energies, are fully defined in Eqs 2–5.

The goal for this model was to include the simplest chemo-mechanical dynamics necessary to investigate the effect of the stochastic time delay between ATP adsorption and ADP/Pi release. The three-state model utilized in this work separates these events into two different state transitions of the cross-bridge cycle. Briefly, the three states of the myosin cross-bridge in our model are (1) myosin not bound to actin, but bound to ADP and Pi (M.ADP.Pi), (2) pre-power stroke state; myosin bound to actin, ADP, and Pi (A.M.ADP.Pi), and (3) post-power stroke state; myosin bound to actin (A.M) (Fig 2). The transitions between these states are stochastic, and are the source of randomness in the behavior of the model. This stochasticity was modeled using a Metropolis Monte Carlo algorithm (details in Section C of S1 Text). The energy landscape of the three states and transitions between them (simplified schematic in Fig 3) was formulated similarly to previous works, except the ATP hydrolysis and detachment of ADP and Pi were accounted for in separate states:

$$\begin{array}{ccc} G_{1} & = & 0 \end{array}$$ (2)

$$\begin{array}{ccc} G_{2} & = & \Delta G_{\text{bind}}+\frac{1}{2}k_{xb}{\left(x_{m}-x_{a}-b_{0}\right)}^{2} \end{array}$$ (3)

$$\begin{array}{ccc} G_{3} & = & \Delta G_{\text{bind}}+\Delta G_{\text{stroke}}-\Delta G_{D,P_{i}\;\text{rel}.}\\ & & +\frac{1}{2}k_{xb}{\left(\left(x_{m}-x_{a}-b_{0}\right)+d_{ps}\right)}^{2}+k_{B}T\;\text{ln}(\frac{\left[\text{ADP}][\text{Pi}\right]}{{\left[\text{ADP}\right]}^{*}{\left[\text{Pi}\right]}^{*}}) \end{array}$$ (4)

$$\begin{array}{ccc} G_{1^{\prime }} & = & -\Delta G_{\text{hyd}}^{*}-k_{B}T\;\text{ln}(\frac{\left[\text{ATP}\right]/{\left[\text{ATP}\right]}^{*}}{\left(\left[\text{ADP}\right]/{\left[\text{ADP}\right]}^{*}\right)\left(\left[\text{Pi}\right]/{\left[\text{Pi}\right]}^{*}\right)}) \end{array}$$ (5)

A reference energy of 0 was set for the free energy of the state 1 (Eq 2). Traveling through each cycle requires the addition of an ATP from the environment, thus we defined state 1’ as the energy baseline of the following new cross-bridge cycle (Eq 5). All parameters were taken from literature (details in Section D of S1 Text), and then fine-tuned to match the physiological duty ratio [31]. Fine tuning of parameters may need to be adjusted within their pre-determined ranges depending on the specific geometry of the system. For a one-myosin system, all variables and parameters are defined in Table 1. The rate constants along with their equilibrium ratios were defined as follows, using the notations from Table 1:

**Table 1:** Table of variables. Variables and the corresponding units or values used in the sarcomere model. Values were selected after a parameter exploration was performed on a range of values pulled from literature from both models and experiments (details in Section D of S1 Text).

Variable (symbol)	Definition	Units or Constant Value
*a*_0_	Rest length of actin spring and distance between adjacent actin nodes in an unstretched thin filament	12.3 nm [15–17, 23, 32, 33]
*m*_0_	Rest length of myosin spring and distance between adjacent myosin nodes in an unstretched thick filament	14.3 nm [15–17, 23, 33–35]
*b*_0_	Rest length of a cross-bridge and the horizontal distance between the base and tip of a myosin head	10 nm [14]
*γ*_0_	Rest length of titin and the distance between the Z-line and thick filament	nm (varies by system)
*d*_*ps*_	Distance myosin head moves during the power stroke with no resistance	7 nm [13, 15, 36]
*k*_*m*_	Stiffness of myosin filament	6060 pN/nm [16, 23]
*k*_*a*_	Stiffness of actin filament	5230 pN/nm [16, 23]
*k*_*xb*_	Stiffness of cross-bridge	3 pN/nm [16, 23, 36]
*k*_*T*_	Stiffness of titin	10 pN/nm [37]
*k*_*F*_	Stiffness of external substrate sarcomere is contracting against	N/*m*
*k*_*B*_ *T*	Thermal energy	4.14 pN ⋅ nm
*β*	*β* = 1/*k*_*B*_*T*	0.241 pN^−1^ ⋅ nm^−1^
Δ*G*_bind_	Free energy drop associated with a myosin head binding to actin	−4*k*_*B*_*T* [12, 21, 38, 39]
Δ*G*_stroke_	Free energy drop associated with the myosin head changing conformation during the power stroke	−4.5*k*_*B*_*T* [12, 21, 22]
$\Delta G_{\text{hyd}}^{*}$	Standard free energy of ATP hydrolysis, includes standard free energy of hydrolysis and chemical potential contributions of hydrolysis substrate and products: ATP, ADP, and Pi at standard conditions i.e. [ATP]* = [ADP]* = [Pi]* = 1M.	13*k*_*B*_*T* [40, 41]
Δ*G*_D,Pi rel._	Free energy associated with the dissociation of ADP and Pi from the actomyosin complex during the power stroke	−14*k*_*B*_*T* [42–48]
*k*_bind_	Rate of myosin binding when there is no distortion in the cross-bridge	800 s^−1^ [12, 16, 21, 22, 49]
*k*_23,*cap*_	Rate of myosin undergoing power stroke when there is no distortion in cross-bridge	30 s−1 [21, 22, 36, 39, 49, 50]
*k*_*ATP*,0_	Rate of reverse hydrolysis of ATP and ATP detachment from myosin	1 × 10^−2^ s^−1^ [44, 51]
*x*_*a*_ or *a*_*i*_	Location of the actin node on the x-axis; *a*_*Z*_ represents the Z-line (x = 0)	nm
*x*_*m*_ or *m*_*i*_	Location of the myosin node on the x-axis (at the Z-line, x = 0); *m*_*M*_ represents the M-line	nm
*x*_*m*_ − *x*_*a*_ − *b*_0_	Cross-bridge displacement; difference between the displacement of a myosin node from the nearest available actin node and cross-bridge rest length. If the cross-bridge is in state 3, the negative of this value is the effective sliding distance (ESD) associated with the power stroke.	nm
*x*_*f*_	Location of the end of the external spring anchor along the x-axis (x = 0 at the Z-line)	nm
*x*_*f*,0_	Rest length of the external spring and distance between the M-line and external spring anchor, *x*_*f*_	100 nm
*K*_*ij*_(*x*)	Chemical equilibrium constant between states *i* and *j*	unitless
*k*_*ij*_(*x*)	Rate constant of transition from state *i* to state *j*	s^−1^

State 1 to State 2:

$$\begin{array}{ccc} K_{12}\left(x_{m},x_{a}\right) & = & \text{exp}\{-\beta [\Delta G_{\text{bind}}+\frac{1}{2}k_{xb}{\left(x_{m}-x_{a}-b_{0}\right)}^{2}]\} \end{array}$$ (6)

$$\begin{array}{ccc} k_{12}\left(x_{m},x_{a}\right) & = & k_{\text{bind}}\text{exp}\{-\beta \frac{1}{4}k_{xb}{\left(x_{m}-x_{a}-b_{0}\right)}^{2}\} \end{array}$$ (7)

$$\begin{array}{ccc} k_{21}\left(x_{m},x_{a}\right) & = & k_{12}/K_{12}=k_{\text{bind}}\;\text{exp}\{\beta [\frac{1}{4}k_{xb}{\left(x_{m}-x_{a}-b_{0}\right)}^{2}+\Delta G_{\text{bind}}]\} \end{array}$$ (8)

State 2 to State 3:

$$\begin{array}{ccc} K_{23}\left(x_{m},x_{a}\right) & = & \text{exp}\{-\beta [\Delta G_{\text{stroke}}-\Delta G_{D,Pi\;rel.}+\\ & & +\frac{1}{2}k_{xb}\left(2\left(x_{m}-x_{a}-b_{0}\right)d_{ps}+d_{ps}^{2}\right)+\\ & & +k_{B}T\;\text{ln}(\frac{\left[\text{ADP}][\text{Pi}\right]}{{\left[\text{ADP}\right]}^{*}{\left[\text{Pi}\right]}^{*}})]\} \end{array}$$ (9)

$$\begin{array}{ccc} k_{23} & = & k_{23,cap} \end{array}$$ (10)

$$\begin{array}{ccc} k_{32}\left(x_{m},x_{a}\right) & = & k_{23}/K_{23}=\\ & = & k_{23,cap}\;\text{exp}\{\beta [\Delta G_{\text{stroke}}-\Delta G_{D,Pi\;rel.}+\\ & & +\frac{1}{2}k_{xb}\left(2\left(x_{m}-x_{a}-b_{0}\right)d_{ps}+d_{ps}^{2}\right)+\\ & & +k_{B}T\;\text{ln}(\frac{\left[\text{ADP}][\text{Pi}\right]}{{\left[\text{ADP}\right]}^{*}{\left[\text{Pi}\right]}^{*}})]\} \end{array}$$ (11)

State 3 to State 1:

$$\begin{array}{ccc} K_{31}\left(x_{m},x_{a}\right) & = & \text{exp}\{-\beta [-\Delta G_{\text{bind}}-\Delta G_{\text{stroke}}-\Delta G_{\text{hyd}}+\Delta G_{D,Pi\;rel.}-\\ & & -\frac{1}{2}k_{xb}{\left(\left(x_{m}-x_{a}-b_{0}\right)+d_{ps}\right)}^{2}-k_{B}T\;\text{ln}(\frac{\left[\text{ATP}\right]}{{\left[\text{ATP}\right]}^{*}})]\} \end{array}$$ (12)

$$\begin{array}{ccc} k_{31}\left(x_{m},x_{a}\right) & = & K_{31}k_{13}=\\ & = & k_{ATP,0}\text{exp}\{-\beta [-\Delta G_{\text{bind}}-\Delta G_{\text{stroke}}-\Delta G_{\text{hyd}}+\Delta G_{D,Pi\;rel.}-\\ & & -\frac{1}{2}k_{xb}{\left(\left(x_{m}-x_{a}-b_{0}\right)+d_{ps}\right)}^{2}-k_{B}T\;\text{ln}(\frac{\left[\text{ATP}\right]}{{\left[\text{ATP}\right]}^{*}})]\} \end{array}$$ (13)

$$\begin{array}{ccc} k_{13} & = & k_{ATP,0} \end{array}$$ (14)

Accordingly, the overall reaction quotient

$$\begin{array}{c} \frac{k_{12}k_{23}k_{31}}{k_{21}k_{32}k_{13}}=\frac{\left[\text{ATP}\right]/{\left[\text{ATP}\right]}^{*}}{\left(\left[\text{ADP}\right]/{\left[\text{ADP}\right]}^{*}\right])\left(\left[\text{Pi}\right]/{\left[\text{Pi}\right]}^{*}\right)}\text{exp}(\frac{1}{k_{B}T}\Delta G_{\text{hyd}}) \end{array}$$ (15)

is consistent with fundamental thermodynamic requirements and involves only the combination of concentrations [ATP]/[ADP][Pi] and hydrolysis free energy Δ*G*_hyd_. At the same time, individual rate constants, *k*_*ij*_, depend only on concentrations of the metabolites relevant to transitions between states *i* and *j*, e.g. *k*_32_ (Eq 11) does not depend on [ATP], while *k*_31_ (Eq 13) does not depend on [ADP] and [Pi].

The solution implementation in MATLAB is discussed in detail in Sections B and C of S1 Text. For all simulations in this investigation, the sarcomere was allowed to spontaneously contract (i.e. no assigned velocity of shortening), with full calcium activation of all binding sites (actin nodes) along the thin filament.

### Validation

The model implementation was verified against the inherent physics of the system. For example, we compared the energy input into the system via ATP hydrolysis to the total elastic potential energy of the springs in the system. Model outputs were compared to those reported in experimental literature, such as ranges of values for ATP consumption, peak force of a single myosin, and force per myosin in larger systems. Independently, reports of rigor concentrations of ATP were compared to predictions made by this model.

## Results

**Fig 4:**
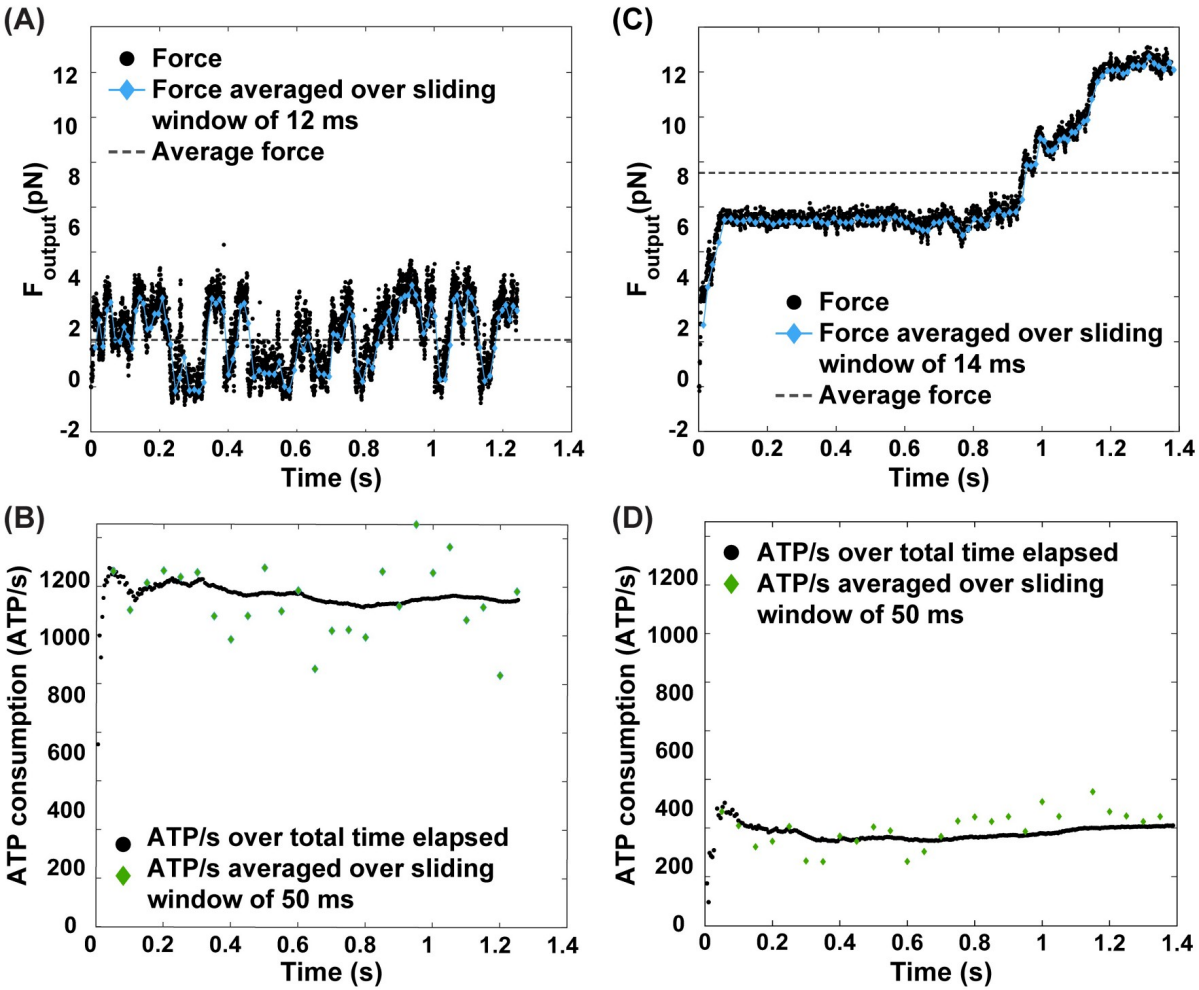
Model Implementation. Single half-sarcomere consisting of 16 myosin and 24 actin nodes under (A-B) normal ([ATP] = 5 mM) and (C-D) reduced ([ATP] = 0.5μM) ATP conditions. (A,C) Force output profile denoted by black circles. Average force denoted by the dashed line. Force averaged over a sliding window of (A) 12 ms and (C) 14 ms denoted by blue lines with blue diamonds. (B,D) ATP consumption rate (molecules/s) denoted by black circles. ATP consumption rate averaged over a sliding window of 50 ms denoted by green diamonds.

Before investigating the impact of the new free energy schema on sarcomere behavior across a range of [ATP] and [ADP][Pi] concentrations, we first validated the model by simulating a single myosin system at the standard normal concentrations for ATP, ADP, and Pi: 5 mM, 0.03 mM, and 3 mM respectively [13, 29, 52, 53]. This simulation revealed a peak force of 3 pN per myosin. This value is consistent with a previous literature range of ∼1–7 pN [32, 54–57]. Next, a half-sarcomere consisting of 16 myosin and 24 actin nodes was simulated (Fig 4A) with the same parameters as a single myosin system (Table 1). The average sarcomeric force output was 2.1 pN (dashed line, Fig 4A), resulting in a time-averaged force per myosin of 0.13 pN. The estimation method described previously for organ-scale contraction [58] was applied to data from other experiments, including muscular thin films (tissue-scale) and traction force microscopy (cell-scale) [59–63], which resulted in the range of forces per myosin in different systems to be 0.04–1 pN. Thus, we conclude that our values of time-averaged force/myosin are within physiologically expected ranges.

To validate the order of magnitude of ATPase activity, ATP consumption rate per myosin was calculated by approximating the density of myosin heads per muscle tissue volume (0.48–1.2 × 10^17^ myosin/cm^3^) from literature estimates of myosin concentration in muscle [64–67]. Based on experimental data from isometrically and concentrically contracting muscle, an ATP consumption estimate would be on the order of 1–120ATP/s per myosin [41, 65–76]. If one takes into account the proportion of myosin hypothesized to actually be participating in contraction [77], an estimate would yield a range of 2–240ATP/s per myosin (further details in Section E of S1 Text). In the model, ATPase activity was then quantified by tracking the number of myosin transitions (per unit time) from state 3 to state 1–the transition that involves the hydrolysis of one ATP molecule (Fig 4B). To avoid biases from the initialization of the contraction simulation, plateau ATP consumption rates were calculated as the mean rate after the rate first exceeds 98% of the maximum consumption rate. The plateau ATP consumption rate of the single 16-myosin sarcomere system was 1400ATP/s (Fig 4B) or about 88ATP/s per myosin, matching our physiological estimates.

Having validated the model, we next demonstrated that there was a significant change to the behavior of the sarcomere when the concentration of [ATP] was changed to 0.5 μM (Fig 4C and 4D), while concentrations of [ADP] and [Pi] were maintained at those found under standard conditions. As can be seen from the force plot (Fig 4C), the sarcomere exhibited rigor like behavior with slow “ratcheting”–characterized by repeated cycles of brief contractile force increases followed by periods of force stagnancy due to lack of ATP. The effect of reduced [ATP] also manifests itself in the ATP consumption rate (Fig 4D), which is significantly reduced compared to that of the system under standard conditions.

**Fig 5:**
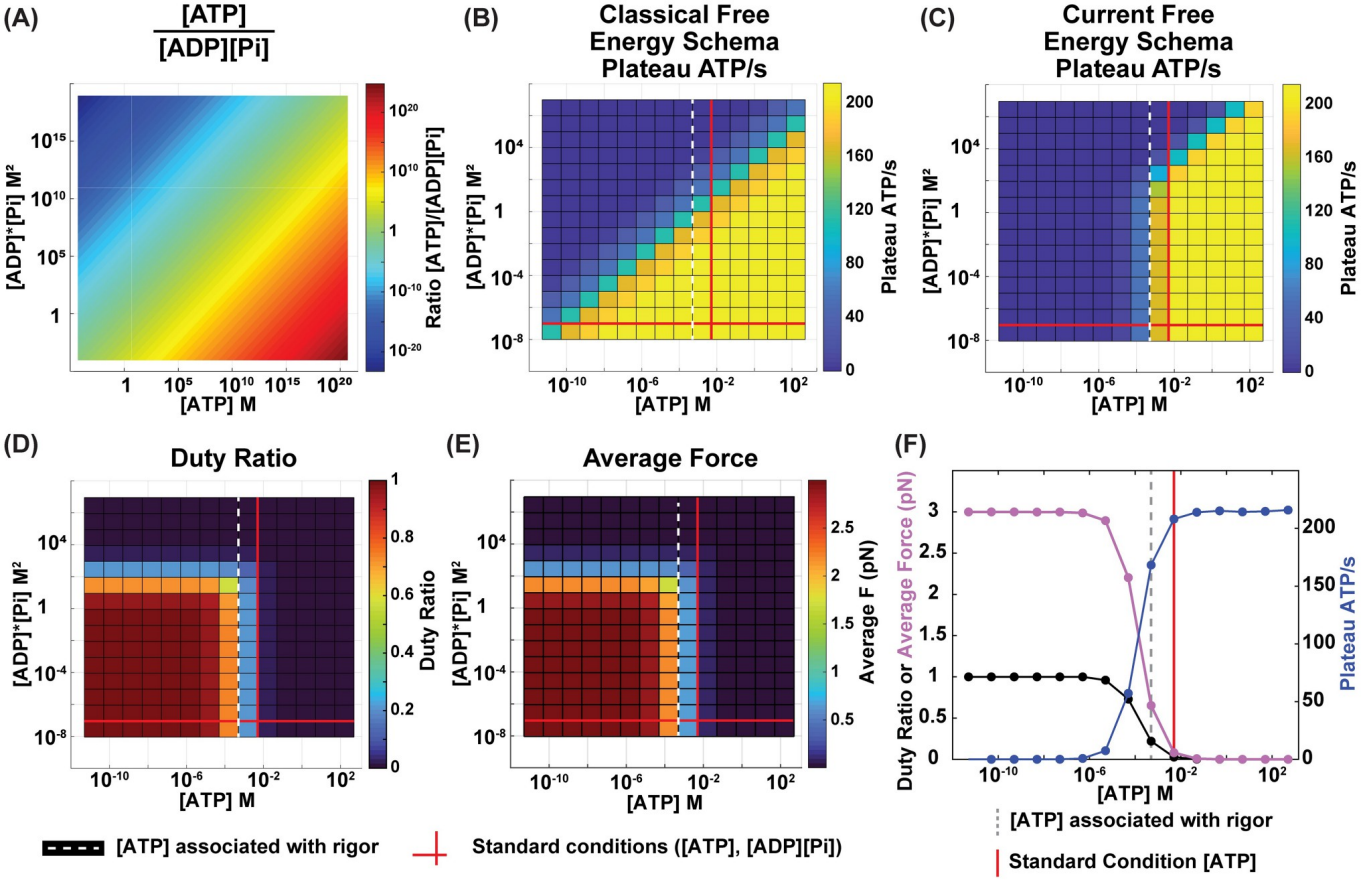
One-myosin system simulations. Comparison of free energy schema in terms of ATP consumption (A) Ratios of [ATP] to [ADP][Pi]. (B) Plateau ATP consumption rate for a one-myosin system where attachment of ATP and detachment of ADP and Pi effectively happen simultaneously, allowing for no time delay between these events. For such a model, the rate kinetics, and thus ATP consumption, depend only the ratio of [ATP]/[ADP][Pi]. (C) Our model’s plateau ATP consumption rate simulation results for a one-myosin system. Note the plot’s asymmetry compared to (B) and the proximity of the standard physiological conditions (bold red lines) to the crossover between regimes. One-myosin simulation results of (D) Duty ratio and (E) Average force for various ([ATP], [ADP][Pi]) combinations. (F) Changes in duty ratio (black), average force output (purple), and plateau ATP consumption rate (blue) at standard physiological [ADP][Pi] and varying [ATP] concentrations. (B-F) Standard physiological conditions are denoted by bold red lines ([ATP] = 5 mM, [ADP][Pi] = 0.09 mM^2^). Dashed white/gray lines denote the the [ATP] concentration associated with the onset of rigor ([ATP] = 0.5 mM) [75, 79]. (C-F) n = 10.

To further explore this, we considered situations with varying values of concentrations of [ATP] and [ADP][Pi]. If all transition rates were to be assumed dependent only upon the ratio [ATP]/[ADP][Pi], all fluctuations in the sarcomere, as well as the average times a myosin head spends in states 1, 2, and 3, would also solely depend on only the ratio [ATP]/[ADP][Pi]. Fig 5A demonstrates how very different values of [ATP] and [ADP][Pi] can have ratios that are the same (equivalent ratios displayed in the same color), resulting in the diagonal symmetry. This point is illustrated in Fig 5B, where we show what the ATP consumption for a single myosin system would look like if state transitions were dependent upon only the ratio [ATP]/[ADP][Pi]. Such a system would have state transition rates that are equivalent as long as the ratio [ATP]/[ADP][Pi] is the same.

The ability of our model to consider separately how [ATP], [ADP], and [Pi] affect the transition rates allowed us to interrogate imbalances in these molecules (Eqs 2–14). Our model demonstrated that, for a single myosin simulation across varying ([ATP], [ADP][Pi]) combinations, there is in fact an asymmetry in ATP consumption that directly results from the appropriate allocation of the overall free energy change of the cross-bridge cycle (Fig 5C). Importantly, the physiological concentration of [ATP] falls within the range of the observed transition region roughly from 10mM to 5 μM (yellow to blue, Fig 5C). This change in regime is also visible in one myosin system simulation outputs for duty ratio (the fraction of time a myosin head spends in state 3 [31, 48, 78]) and average force (Fig 5D–5F).

Furthermore, the ATP concentration at which the system begins to exhibit rigor-like characteristics (dashed white/gray line, Fig 5C–5F), indicated by a rise in duty ratio, is consistent with the one experimentally observed with the onset of rigor ([ATP] ≤ 0.5 mM) [75, 79]. Notably, the free energy schema utilized by this model was constructed completely independent of any experimental results on rigor-inducing concentrations of [ATP]. Therefore, the alignment between our model’s predictions and experimental values acts as an independent validation of the proposed free energy schema.

**Fig 6:**
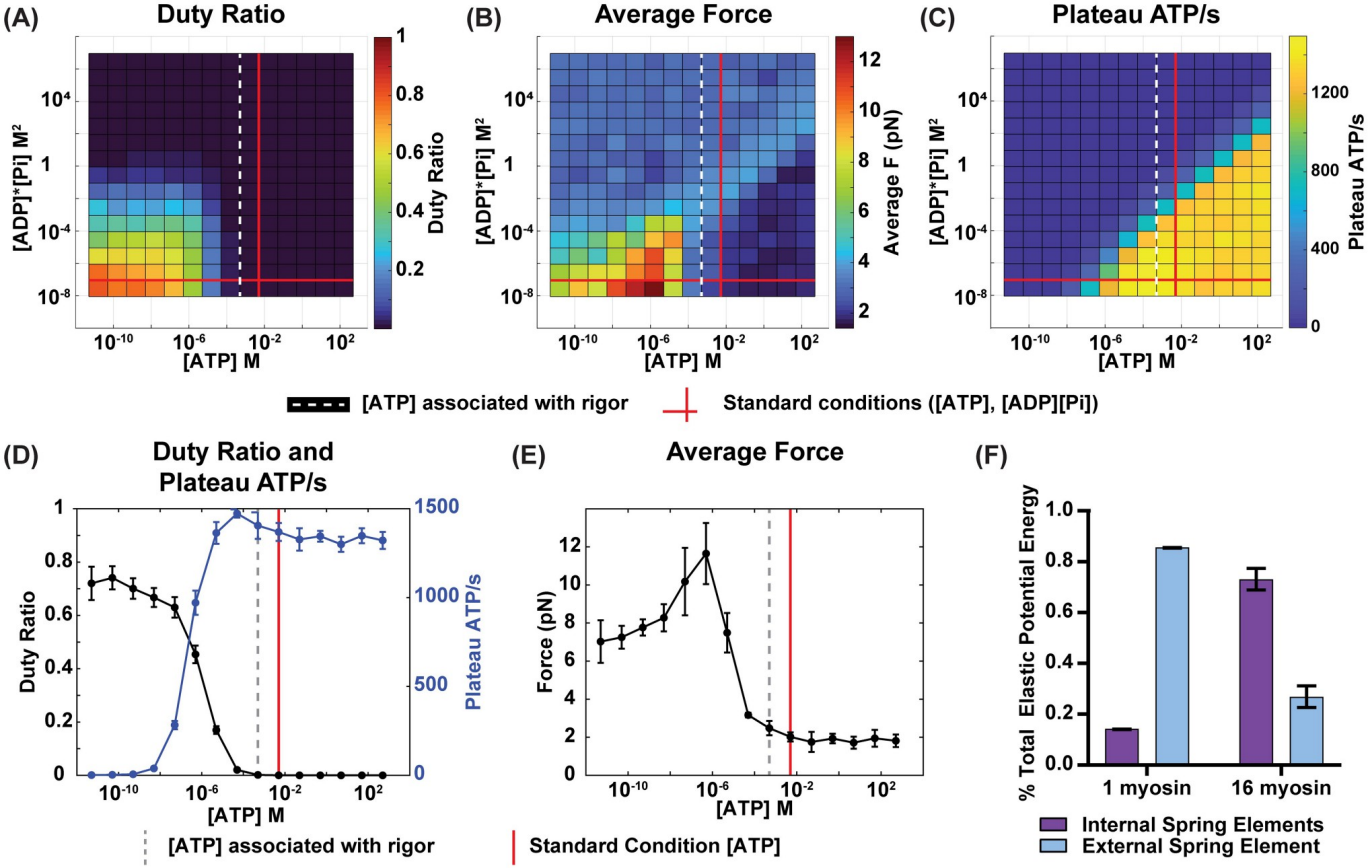
16-myosin system simulations. Results for 16-myosin simulations (n = 10) showing (A,D) Duty ratio, (B,E) Average force, and (C, D) Plateau ATP consumption rate. Average force and plateau ATP consumption rate are reported as per whole sarcomere. (D) Duty ratio and ATP consumption rate and (E) Average force at standard physiological [ADP][Pi] and varying [ATP] concentrations. (A-E) Standard physiological concentrations for [ATP] and [ADP][Pi] conditions are denoted by the bold red lines ([ATP] = 5 mM, [ADP][Pi] = 0.09 mM^2^). Dashed white/gray lines represent literature values of [ATP] concentrations where the onset of rigor has been observed experimentally ([ATP] = 0.5 mM) [75, 79]. (F) Percent of total elastic potential energy in the spring elements internal to the sarcomere (i.e. actin, myosin, titin, and cross-bridges) and external to the sarcomere (i.e. external spring).

Next, we examined duty ratio, average force, and plateau ATP consumption rate for a simulation of a 16-myosin half-sarcomere model with the same parameters as a single myosin system (Table 1) across varying ([ATP], [ADP][Pi]) combinations and qualitatively observed similar asymmetry in duty ratio, average force, and plateau ATP consumption rates (Fig 6A–6C). For example, in the region with lower than normal [ATP], and low to normal [ADP][Pi], multi-myosin half-sarcomere systems will ratchet up the thin filament to a greater degree of shortening (Figs 4C and 6A–6C). This ratcheting behavior is what is expected for muscle that can contract but not relax. While in other regions, such as physiological-adjacent conditions, the system’s force output fluctuates more naturally (Figs 4A and 6A–6C). In examining the regime change associated with the onset of rigor at normal [ADP][Pi] (0.09 mM^2^), we noted a shift from where the transition is reported physiologically by one order of magnitude (Fig 6D and 6E). We hypothesized that this is likely caused by the more complex internal tensions of a multi-myosin system shifting the duty ratio to lower than normal (Fig 6A and 6D). The first step in testing this hypothesis was to evaluate whether internal tensions can change expected sarcomere deformations. Indeed, the effective sliding distance (ESD) of the one-myosin system simulation is actually less than the prescribed *d*_*ps*_ by approximately 1 nm. Moreover, when comparing one-myosin and multi-myosin systems, the system with more myosin has a larger percentage of the its total elastic potential energy stored within the sarcomere’s internal spring elements (i.e. actin, myosin, titin, bound cross-bridges) as opposed to to the external spring element (i.e. the substrate sarcomere is contracting against), also impacting the ESD (Fig 6F).

To further test this hypothesis, we created a powerful tool to explore the effect of different parameters by analytically calculating the duty ratio, average force, and plateau rate of ATP consumption for a one myosin system using the rate constant equations that govern state transitions (Eqs 6–14):

$$\begin{array}{ccc} \text{Duty}\;\text{Ratio} & = & \frac{P_{3}}{P_{1}+P_{2}+P_{3}} \end{array}$$ (16)

$$\begin{array}{ccc} \text{Avg}.\;\text{Force} & = & \left\{\text{Duty}\;\text{Ratio}\right\}*\left\{\text{Peak}\;{\text{Force}}_{\text{one-myosin},\;\text{sim}.}\right\} \end{array}$$ (17)

$$\begin{array}{ccc} \text{ATP}\;\text{consumption}\;\text{rate} & = & \frac{k_{31}P_{3}-k_{13}P_{1}}{P_{1}+P_{2}+P_{3}} \end{array}$$ (18)

where

$$\begin{array}{ccc} P_{1} & = & k_{23}k_{31}+k_{21}\left(k_{32}+k_{31}\right) \end{array}$$ (19)

$$\begin{array}{ccc} P_{2} & = & k_{31}k_{12}+k_{32}\left(k_{12}+k_{13}\right) \end{array}$$ (20)

$$\begin{array}{ccc} P_{3} & = & k_{12}k_{23}+k_{13}\left(k_{21}+k_{23}\right) \end{array}$$ (21)

and Peak Force_one-myosin,sim._ ≈ 3 pN is the peak force exerted by a myosin cross-bridge in simulation of a one-myosin system.

**Fig 7:**
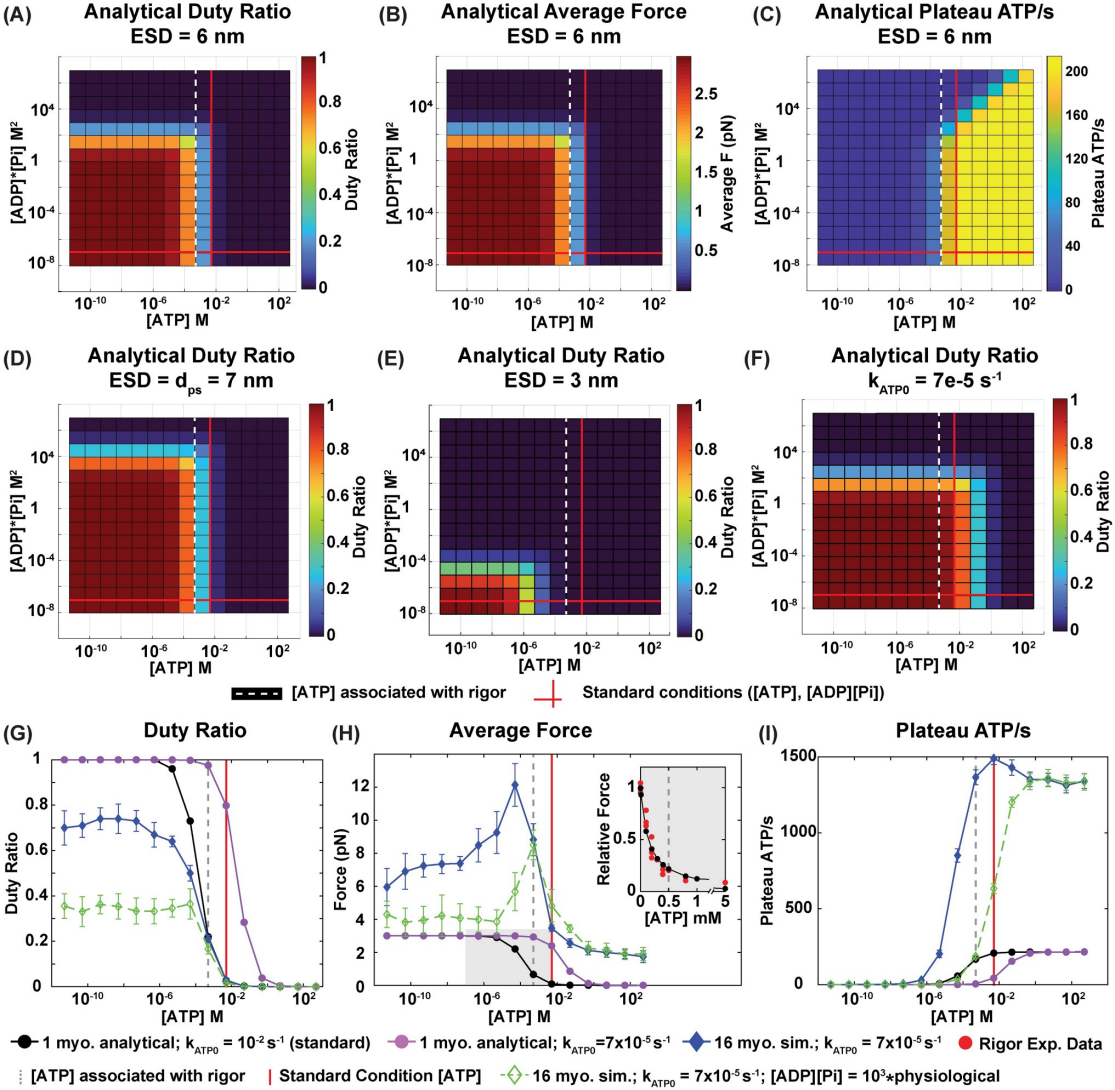
Analytical results for a one-myosin system and varying parameters. (A) Analytically calculated duty ratio (Eq 16) where the power stroke’s effective sliding distance (ESD) = 6 nm. (B) Analytically calculated average force (Eq 17) where ESD = 6 nm. (C) Analytically calculated ATP consumption rate (Eq 18) where ESD = 6 nm. (D) Analytically calculated duty ratio where ESD = *d*_*ps*_ = 7 nm, where 7 nm is the power stroke distance against no resistance. This causes an upward and rightward shift in the plot. (E) Analytically calculated duty ratio where ESD = 3 nm. This causes a downward and leftward shift in the plot. (F) Analytically calculated duty ratio where ESD = 6 nm and *k*_*ATP*,0_ = 7 × 10^−5^ s^−1^. This causes a rightward shift in the plot. (A-I) Standard physiological concentrations for [ATP] and [ADP][Pi] conditions are denoted by the bold red lines ([ATP] = 5 mM, [ADP][Pi] = 0.09 mM^2^). Dashed white/gray lines denote the [ATP] concentration associated with the onset of rigor ([ATP] = 0.5 mM) [75, 79]. Effect of reducing *k*_*ATP*,0_ on one-myosin analytical and 16-myosin simulation (n = 10) values for (G) Duty ratio, (H) Average force, and (I) Plateau ATP consumption rate at standard physiological [ADP][Pi] or increased [ADP][Pi] and varying [ATP] concentrations. Reducing *k*_*ATP*,0_ causes a rightward shift in all of the plots. Increasing environmental [ADP][Pi] by a factor of 10^3^ alters 16 myosin system behavior. Average force and plateau ATP consumption rate are reported as per whole sarcomere. (H, inset) The one-myosin analytical system’s predictions of average force compared to muscle strip data (red circles) adapted from White [79]. Data are normalized to force under complete rigor, at [ATP] = 0.1 μM for the analytical system and [ATP] = 0mM for experimental data.

The analytical outputs for a single myosin system (Fig 7A–7C) align with the simulation results (Fig 5C–5E) if the effective sliding distance, ESD = 6 nm. To accomplish this, we replaced the cross-bridge displacement term (Table 1) in Eqs 9, 11–13 and 16–21 as follows: (*x*_*m*_ − *x*_*a*_ − *b*_0_) = −ESD = − 6 nm. In contrast, the match is not as close when the ESD is assumed to be the same as prescribed: ESD = *d*_*ps*_ = 7 nm (Fig 7D). This implies that if our hypothesis is correct, smaller ESDs would lead to analytical solutions that mimic the 16-myosin simulation (Fig 6A), which was indeed observed at ESD ≈ 3 nm (Fig 7E).

Although the effective sliding distance cannot be prescribed in a simulation, the phenomena of internal tensions can be manipulated by extending the duration each myosin head remains attached to actin, controlled by adjusting the *k*_*ATP*,0_ parameter in Eqs 13, 14 and 16–21 while holding all other parameters as in Table 1. Indeed, by reducing *k*_*ATP*,0_ from 10^−2^] s^−1^ to 7 × 10^−5^ s^−1^, we observe a rightward shift in the model’s outputs (Fig 7F–7I). A 16-myosin sarcomere simulated with this shift enables the system to exhibit the same average duty ratio among its cross-bridges at physiological [ATP], [ADP][Pi] as a one-myosin system with the original *k*_ATP,0_ (Fig 7G). Excitingly, this realignment towards a physiological duty ratio is concurrent with the shift in the 16-myosin system’s rigor behavior, matching physiological expectations (Fig 7G–7I). A feature exclusive to a shorter ESD system, e.g. the 16-myosin system, is its sensitivity to [ADP][Pi] changes near physiological conditions. For example, if environmental [ADP][Pi] is increased by a factor of 10^3^, there are significant shifts in physiological and rigor associated force outputs and ATP consumption (dashed green line, Fig 7H and 7I).

## Discussion

A sarcomere is the fundamental unit of muscle contraction, and modeling its behavior can provide insight into the mechanisms of muscle function. The model in this study bridges the gap between stochastic-mechanical sarcomere models and a novel cross-bridge cycle kinetic schema that considers [ATP], [ADP], and [Pi] in their relevant state transitions, conferring it the advantages of both types of models (Fig 3). The rate constant definitions in our model were derived with few underlying assumptions, enabling them to be simpler and more direct than in previous models (Eqs 6–12 and 15), while also effectively capturing how changes in free energy–and consequently kinetic rates–arise from independent perturbations in either [ATP] or [ADP][Pi].

Utilizing our new kinetic schema, we showed that, for each geometry, as long as the duty ratio remained physiological at standard [ATP], [ADP][Pi] levels, it was possible to predict the concentration at which the onset of rigor is expected (Figs 5C–5F, 7A–7C, 7G–7I), with the analytical solution agreeing remarkably with experimental data [79] (Fig 7H, inset). This approach allowed us to observe how shifts in sarcomere behavior could arise from changes in internal mechanics or kinetic adjustments, demonstrated in this study by varying the effective sliding distance or adjusting the parameter *k*_*ATP*,0_. The results suggest that *k*_*ATP*,0_ is a key parameter in fine-tuning the model’s accuracy and relevance in more complex systems (Fig 7G–7I). The backward transition that *k*_*ATP*,0_ governs may be even lower for 3D systems, whose internal tensions are expected to be even more intricate than our 16-myosin system’s (Fig 6F and contrasting Fig 6D and 6E with Fig 7G–7I). Modifying *k*_*ATP*,0_ to account for internal tensions is consistent with theoretical considerations of cross-bridge cycling under increased internal tensions [80, 81] and experimental insights of myosin binding kinetics’ role in muscle function [82], and is thus a valid means of maintaining model fidelity when investigating increasingly complex muscular systems. Therefore, by simple tuning of the duty ratio, a key feature of this model, the kinetic schema can be applied to studies of different myosin classes and isoforms, conditions characterized by altered muscle energetics, and muscle adaptation to energy stress.

Consistent with the results of our model (Figs 4–7), it is well documented that a sarcomere’s environmental conditions can lead to increased myosin binding to actin (e.g. low-ATP concentrations, high-ADP concentrations, or rigor conditions) or decreased myosin binding to actin (e.g. high-Pi concentration or myosin inhibitors) [83–86], as is evident in many pathologies [1–5]. Beyond the scale of myosin binding, changes in concentrations of any one of these metabolites has shown varied effects on force metrics of muscle fibers [87–89], in alignment with our results on force output (Fig 7B and 7H). Even in non-pathological states, such as during muscle fatigue, [ADP] and [Pi] concentrations can increase significantly (20 to 300-fold and 6 to 10-fold respectively–an increase up to the order of 10^3^ in [ADP][Pi] [52, 90–94]), leading to changes in force output and energetics, which our model can explore (Fig 7H and 7I). Taken together, the existence of these conditions where the concentrations of these metabolites move independent from one another necessitates contraction models that are capable of interrogating the effects of imbalances in the [ATP]/[ADP][Pi] ratio based on individual concentrations.

As this model was intentionally made to be simple, it does not include additional intermediate states of the cross-bridge cycle or more complex characteristics of a real sarcomere. For example, according to recent discoveries, the super-relaxed (SRX) state of myosin is important due to its potential role in optimizing sarcomeric energy utilization [77, 95, 96]. Thus, SRX states may need to be included in future iterations of the model as it has been suggested that altered concentrations of environmental [ADP] may cause strain-mediated destabilization of the SRX population in sarcomeres [77, 95]. While the phenomenological implementation of this SRX state has been previously modeled [24, 97], to include this additional cross-bridge state mechanistically within our model would require more experimental data.

The force traces analyzed in this study observe a half-sarcomere system as it contracts against an external spring, so there is no prescribed velocity of shortening or isotonic shortening (Fig 4A). This implementation closely mimics the experimental conditions under which cellular and tissue muscle mechanics are studied, such as in traction force microscopy and muscular thin films [59–63]. Therefore, the model can be paired with “heart-chip” experiments that explore the effect of hypoxia [98] or other altered [ATP], [ADP], [Pi] conditions to predict reductions in contractility. Similarly, in the future the kinetic schema developed here can be hybridized with prescribed velocity contraction models to explore how power density of the muscle changes with varying [ATP] [99].

## Conclusion

This simple model adequately captured the geometries, mechanics, and kinetics necessary for investigating the chemical and mechanical outputs of sarcomeric force generation while also providing the flexibility to interrogate sarcomeric response to alterations in mechanical and kinetic parameters. The novel approach to the allocation of free energies within this model enabled evaluation of sarcomeric outputs in response to changes in [ATP], [ADP], and [Pi] concentrations that would be otherwise misrepresented. The associated analytical solution for the one-myosin system is a powerful tool to explore how different parameters influence stochastic-mechanical behavior based on how these parameters affect the analytical system. The gained insights not only validate the utility of our model but also establish a solid foundation for future experimental explorations aimed at targeting muscular disorders at a molecular level. Since our model possesses geometric and mechanical elements generally consistent with those of previous models, our novel kinetic schema is an easily integrated augmentation that will lead to this work’s increased relevance as a tool to interrogate energy utilization and force generation of sarcomeres under a variety of ATP, ADP, and Pi environmental conditions.

## Supporting information

S1 Text This file contains S1 Text Sections A–E with details on model derivation, implementation, and parameter exploration, and further citations supporting parameter and method selection. (PDF)
