# Supplementary material for: A novel kinetic model to demonstrate the independent effects of ATP and ADP/Pi concentrations on sarcomere function
Source: PLoS Comput Biol. 2024 Aug 5;20(8):e1012321. doi: 10.1371/journal.pcbi.1012321 (PMC11326600; doi:10.1371/journal.pcbi.1012321)
Supplement: S1 Text — (PDF) [file pcbi.1012321.s001.pdf]

S1 Supporting Information for "A Novel Kinetic Model to Demonstrate the Independent Effects of ATP and ADP/Pi Concentrations on Sarcomere Function"

Andrew Schmidt, Alexander Y. Grosberg, Anna Grosberg

A. Description of Geometry

The rest length of an actin spring, and thus the distance between actin nodes when the half-sarcomere is at rest, was set to 12.3 nm for all simulations. The rest length of a myosin spring, and thus the distance between myosin nodes when the half-sarcomere is at rest, was set to 14.3 nm for all simulations. The rest length of a myosin cross-bridge was set to 10 nm during states 1 and 2, but is reduced by the length of the power stroke (7 nm) following the conformational change associated with the detachment of ADP and Pi (new rest length = 3 nm). The spacing values are consistent with physiological values of vertebrate striated muscle [1–5]. However, we collapse the three-dimensional array of myosin cross-bridges and actin binding sites to a one-dimensional set of collinear nodes.

B. Detailed Explanation of Mechanics

The following is the set of force balance equations for the 4-node system ($N_m = 2$ and $N_a = 2$). The external spring element (k_F) is bound from the M-line to a fixed location external to the half-sarcomere. The equations below are constructed such that positive force is towards the right (Fig 1). Myosin node #1 is bound to actin node #1 (m_1

bound to a_1) and myosin node #2 is bound to actin node #2 (m_2 bound to a_2).

$$\text{Z-line node:} \quad k_T\left(m_1 - a_Z - \gamma_0\right) + k_a\left(a_1 - a_Z - a_0\right) = 0 \tag{S1}$$

$$\text{node } a_1\text{:} \quad k_a\left(a_2 - a_1 - a_0\right) - k_a\left(a_1 - a_Z - a_0\right) + k_{xb}\left(m_1 - a_1 - b_0\right) = 0 \tag{S2}$$

$$\text{node } a_2\text{:} \quad -k_a\left(a_2 - a_1 - a_0\right) + k_{xb}\left(m_2 - a_2 - b_0\right) = 0 \tag{S3}$$

$$\text{node } m_1\text{:} \quad -k_T\left(m_1 - a_Z - \gamma_0\right) + k_m\left(m_2 - m_1 - m_0\right) - k_{xb}\left(m_1 - a_1 - b_0\right) = 0 \tag{S4}$$

$$\text{node } m_2\text{:} \quad k_m\left(m_M - m_2 - m_0\right) - k_m\left(m_2 - m_1 - m_0\right) - k_{xb}\left(m_2 - a_2 - b_0\right) = 0 \tag{S5}$$

$$\text{M-line node:} \quad k_F\left(x_f - m_M - x_{f,0}\right) - k_m\left(m_M - m_2 - m_0\right) = 0 \tag{S6}$$

The following example of vectors $\boldsymbol{P}$, vector $\boldsymbol{V}$, and matrix $\boldsymbol{K}$ represent the linear equations for a 4-node system where there are no cross-bridges binding the actin and myosin filaments. Force output is determined by the external spring element bound to the M-line of the half-sarcomere. The titin element binds the Z-line to the first myosin node, and its rest length is denoted by γ_0. For simplicity, we assume titin exerts a passive force when the half-sarcomere is stretched beyond its rest length, but does not exert a compressive force when the half-sarcomere is shortened. A spring constant of 10 pN/nm was chosen for titin because only very small stretching deformations are expected in this model [6]. If the model is to be utilized with large stretches of the sarcomere from its rest length, then a nonlinear spring constant for k_T should be used instead [6].

$$\boldsymbol{KP} = \boldsymbol{V} \tag{S7}$$

$$\begin{bmatrix} -2k_a & k_a & 0 & 0 & 0 \\ k_a & -k_a & 0 & 0 & 0 \\ 0 & 0 & -k_T - k_m & k_m & 0 \\ 0 & 0 & k_m & -2k_m & k_m \\ 0 & 0 & 0 & k_m & -k_m - k_F \end{bmatrix} \begin{bmatrix} a_1 \\ a_2 \\ m_1 \\ m_2 \\ m_M \end{bmatrix} =$$

$$= \begin{bmatrix} -k_a a_Z \\ -k_a a_0 \\ k_m m_0 - k_T(a_Z + \gamma_0) \\ 0 \\ -k_m m_0 - k_F\left(x_f - x_{f,0}\right) \end{bmatrix} \tag{S8}$$

Equation S7 can be re-written for the situation where the cross-bridges are engaged between nodes a_1 and m_1 and between nodes a_2 and m_2. Both cross-bridges are in their bound, pre-power stroke conformation. Force output is determined by the external spring element bound to the M-line of the half-sarcomere:

$$\begin{bmatrix} -2k_a - k_{xb} & k_a & k_{xb} & 0 & 0 \\ k_a & -k_a - k_{xb} & 0 & k_{xb} & 0 \\ k_{xb} & 0 & -k_T - k_m - k_{xb} & k_m & 0 \\ 0 & k_{xb} & k_m & -2k_m - k_{xb} & k_m \\ 0 & 0 & 0 & k_m & -k_m - k_F \end{bmatrix} \begin{bmatrix} a_1 \\ a_2 \\ m_1 \\ m_2 \\ m_M \end{bmatrix} =$$

$$= \begin{bmatrix} -k_a a_Z + k_{xb} b_0 \\ -k_a a_0 + k_{xb} b_0 \\ k_m m_0 - k_{xb} b_0 - k_T(a_Z + \gamma_0) \\ -k_{xb} b_0 \\ -k_m m_0 - k_F\left(x_f - x_{f,0}\right) \end{bmatrix} \tag{S9}$$

The following equations describe a binding scheme identical to the previous set of equations. However, node m_1 is now in state 3, its post-power-stroke, high force bearing

state. This state is characterized by a conformational change, which changes the rest length of the m_1 myosin head. This change manifests as an adjustment in the b_0 terms in the $\boldsymbol{V}$ matrix for the nodes involved in the state 3 cross-bridge i.e. a_1 and m_1, changing these terms to include $(b_0 - d_{ps})$ instead, where d_{ps} is the length of the power stroke. The power stroke in this model generates force in the positive x-direction:

$$\boldsymbol{P} = \boldsymbol{K}^{-1}\boldsymbol{V} \tag{S10}$$

$$\boldsymbol{P} = \begin{bmatrix} a_1 \\ a_2 \\ m_1 \\ m_2 \\ m_M \end{bmatrix} \tag{S11}$$

$$\boldsymbol{K} = \begin{bmatrix} -2k_a - k_{xb} & k_a & k_{xb} & 0 & 0 \\ k_a & -k_a - k_{xb} & 0 & k_{xb} & 0 \\ k_{xb} & 0 & -k_T - k_m - k_{xb} & k_m & 0 \\ 0 & k_{xb} & k_m & -2k_m - k_{xb} & k_m \\ 0 & 0 & 0 & k_m & -k_m - k_F \end{bmatrix} \tag{S12}$$

$$\boldsymbol{V} = \begin{bmatrix} -k_a a_Z + k_{xb}(b_0 - d_{ps}) \\ -k_a a_0 + k_{xb} b_0 \\ k_m m_0 - k_{xb}(b_0 - d_{ps}) - k_T(a_Z + \gamma_0) \\ -k_{xb} b_0 \\ -k_m m_0 - k_F\,(x_f - x_{f,0}) \end{bmatrix} \tag{S13}$$

C. Implementation of Kinetics

The stochastic nature of the three-state cross-bridge cycle used in this study was modeled using a Monte Carlo algorithm, more specifically, we implemented an algorithm similar to Gillespie [7]. To describe our algorithm, we first have to define all rate constants, and for that we have to define all relevant free energies (see also Fig 3 and Equations 2-5 in the main text).

State 1 was assigned a reference energy of 0 (Equation 2). The remaining free energy

profiles of state 2 and state 3 are parabolic in relation to the distortion $(x_m - x_a - b_0)$ of the cross-bridge in those states, where x_m represents the x-coordinate of the myosin node, x_a represents the x-coordinate of the associated actin node (nearest, unoccupied node), and b_0 represents the rest length of a cross-bridge. ΔG^*_{hyd} is the standard free energy of ATP hydrolysis. The power stroke distance is represented by d_{ps}, k_B is the Boltzmann constant, T is absolute temperature, assumed to be room temperature at 300K. Under standard conditions, $[\text{ATP}]^* = [\text{ADP}]^* = [\text{Pi}]^* = 1$ M. The normal concentrations for each of these molecules used in this study are: $[\text{ATP}] = 5\,\text{mM}$, $[\text{ADP}] = 0.03\,\text{mM}$, and $[\text{Pi}] = 3\,\text{mM}$ [8–11]. Remaining variables are defined in Table 1. Rate constants of transitions are detailed in Equations 6-13. Consistent with basic principles, the overall reaction quotient,

$$\frac{k_{12}k_{23}k_{31}}{k_{21}k_{32}k_{13}} = \frac{[\text{ATP}]}{[\text{ADP}][\text{Pi}]} \exp\left(\beta \Delta G_{\text{hyd}}\right) \tag{S14}$$

depends only on the combination of concentrations [ATP]/[ADP][Pi] and hydrolysis free energy ΔG_{hyd}, while individual rate constants depend on concentrations beyond this usual “mass action” combination.

Having defined rate constants, we advance time in (uneven!) steps δt, and the probability of the $i \to j$ transition during one such time step should be $P_{ij} = k_{ij}\delta t$. We have to realize that any one such transition in the many-myosin system affects rates of all possible subsequent transitions. This is because of the elastic coupling between myosin heads, expressed in the transition rates’ dependencies on the potential energies and coordinates, x. Therefore, to faithfully represent the correct Poisson statistics of the molecular transitions, it is imperative to choose time step δt so small that only one transition can occur during that time step. To achieve this while avoiding the computationally prohibitive slowness of the algorithm, we vary time steps as follows (see Fig A).

The main idea is to choose the time step such as to limit the chance of an “uneventful” time step, when no transition occurs. We, therefore, choose (arbitrarily) the no-transition probability as a parameter, $P_{\text{no transition}}$; we chose $P_{\text{no transition}} = 0.1$. In other words, some transition should occur with probability $1 - P_{\text{no transition}} = 0.9$. With this in mind, we sum up all the rates of all possible transitions from a current state and

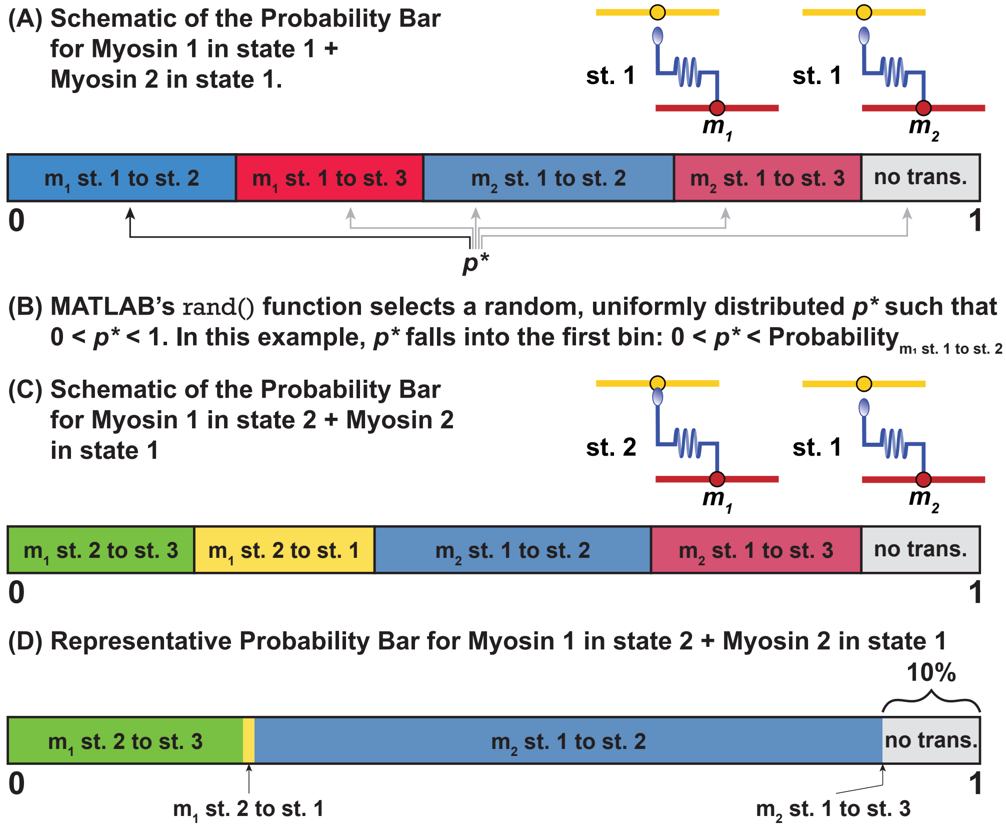

Fig A. Probability bar depicting implementation kinetics using Monte Carlo algorithm. (A) Schematic of how the probability bar may appear for a 2-myosin system where both myosin are in state 1. Half-sarcomere schematic depicts the physical representation of this system. (B) A random number p^* is pulled from a uniform distribution between (0, 1). In this example, p^* falls into the first bin of the probability bar, indicating a forward transition of m_1 from state 1 to state 2. (C) Schematic of the probability bar following this transition, where m_1 is now in state 2 and m_2 is still in state 1. (D) Simulation-representative probability bar of the system above. The size of the probability bins are proportional to their true values as seen in a system where cross-bridges are perfectly aligned with an actin node i.e. there is no deformation of the cross-bridge springs. Note that the probability of no transition occurring is 10% for every time step, regardless of the rate constants' values.

find the next time step as follows:

$$\Sigma_{\text{rates}} = \sum_{n=1}^{m_{\text{total}}} \left[k_{ij}^{n} + k_{ik}^{n} \right] \ , \tag{S15}$$

$$\delta t = \frac{1 - P_{\text{no transition}}}{\Sigma_{\text{rates}}} \ . \tag{S16}$$

Here m_{total} is the number of myosin heads in the system; k_{ij}^{n} and k_{ik}^{n} are, respectively, forward and backward rates of myosin number n, which presently is in the state i.

Then, the probability of $i \to j$ transition for myosin n is expressed as

$$P_{ij}^n = k_{ij}^n \delta t \ . \tag{S17}$$

Knowing this, we separate the interval $(0, 1)$ into $2 * m_{\text{total}} + 1$: two intervals for each myosin with lengths P_{ij}^n and P_{ik}^n, plus one interval of length $P_{\text{no transition}}$. We then generate a random number p^* from a uniform distribution between 0 and 1 using MATLAB's internal `rand()` function (reshuffling random number generator at the start of each simulation using the `rng('shuffle')` function). Depending on the value of this random number, we choose the transition to perform.

Note that although p^* is uniformly distributed between 0 and 1, it does not represent the probability distribution of state transitions. The latter is dictated by the rate constants and, therefore, changes with internal deformations of the sarcomere model.

Probabilities of state transitions occurring are calculated as the product of the rate constant and time step (Equation S17). After state transitions are defined by the Monte Carlo algorithm, we assumed mechanical equilibrium is achieved rapidly relative to the time taken to transition between states. The position of each node within the lattice was calculated by solving a detailed force balance at each node (Equation S10). The formulation of the model is flexible enough to allow for the addition of more actin binding sites (actin nodes) and cross-bridges (myosin nodes).

D. Parameter Exploration

Parameter exploration was performed on this sarcomere model to determine an appropriate set of values for the rate constants. The output of the model to which the parameters were fit was the myosin duty ratio, which in striated muscle is on the order of 0.05 [12–15]. The myosin duty ratio is defined as the proportion of the cross-bridge cycle that the motor domain of the myosin is strongly bound to the actin filament [12–14]. For this parameter exploration, the duty ratio was calculated analytically from the equilibrium constants of each individual cross-bridge transition step (Equations 16, 19-21). These analytical values were found to match those achieved via simulation (Fig 5). Value ranges for the rate constants were taken from previous models and physiological values from literature. Literature values for these parameters

can be found in Table 1. These potential parameter values were varied in a systematic manner to explore the full range of possibilities for each parameter. To determine the optimal parameter values, the model was run for each set of three parameters and the resulting myosin duty ratio was calculated (Fig B-D). The set of parameter values that resulted in the best fit to the duty ratio was selected as the optimal set. The selected parameters for the simulations in this study were well within the blue region of the plots in Fig B-D, meaning that the results of the model are not sensitive to slight parameter perturbations.

The task of this study was not to find optimized sets of model parameters that will apply to all scenarios, but rather to demonstrate how outputs of the model change in response to different geometries and metabolite conditions under a single set of parameters. Thus, parameter values that were chosen for the model were on the order or within physiological ranges with reasonable accuracy. A sensitivity analysis was performed where small perturbations in all of the parameter values were analyzed. The model was found to be robust to such perturbations for the outputs of duty ratio, ATP consumption rate, and average force output. Furthermore, because literature reported values of duty ratio exist within a range (3-5%), the model's parameters can be adjusted in ways to achieve different duty ratios or geometries associated with different muscle types (e.g. fast, slow, cardiac), myosin classes, and species [3, 13, 14]. Similarly, mechanical parameters can also be adjusted in order to model sarcomeres from different physiology. For example, thick filament stiffness varies between different species, and this parameter can be adjusted to match these differences between species [16]. In another example, equilibrium constants between cross-bridge states, and thus free energy changes between states, also vary between myosin isoforms [14, 17–20].

E. Estimation of ATP Consumption Rate

Calculations described in the main text determined that contracting muscle has an ATP consumption rate on the order of 2-240 ATP/s per myosin. The density of myosin heads in muscle tissue was derived from literature estimates of myosin concentration in muscle (0.08-0.2 mM) [21–24]. Many factors can affect the ATP consumption rate of contracting muscle, including temperature, degree of calcium activation, velocity of

shortening, substrate concentrations, myosin isoform, muscle geometry (e.g. lattice spacing, stretch), and species [3, 21, 22, 25–29]. Additionally, literature reports of how many myosin actually participate in contraction (50-60% of myosin are in the SRX state and do not participate in contractility) was also taken into consideration [30]. Lastly, some of the experiments from which estimates of ATP consumption were taken were conducted at lower than physiological temperatures and non-maximal calcium activation. Increased temperature and activation of thin filament binding sites would further increase ATPase rates in these systems. The goal of this study concerning ATP consumption was not to optimize it for all situations, but to observe how this activity varies under different conditions.

References

1. Molloy JE, Burns JE, Sparrow JC, Tregear RT, Kendrick-Jones J, White DC. Single-Molecule Mechanics of Heavy Meromyosin and S1 Interacting with Rabbit or Drosophila Actins Using Optical Tweezers. Biophysical Journal. 1995;68(4 Suppl):298S–305S.

2. Huxley HE, Stewart A, Sosa H, Irving T. X-Ray Diffraction Measurements of the Extensibility of Actin and Myosin Filaments in Contracting Muscle. Biophysical Journal. 1994;67(6):2411–2421.

3. Craig R, Woodhead JL. Structure and Function of Myosin Filaments. Current Opinion in Structural Biology. 2006;16(2):204–212. doi:10.1016/j.sbi.2006.03.006.

4. AL-Khayat HA, Morris EP, Kensler RW, Squire JM. Myosin Filament 3D Structure in Mammalian Cardiac Muscle. Journal of Structural Biology. 2008;163(2):117–126. doi:10.1016/j.jsb.2008.03.011.

5. Holmes KC, Trentham DR, Simmons R, Lombardi V, Piazzesi G, Reconditi M, et al. X-Ray Diffraction Studies of the Contractile Mechanism in Single Muscle Fibres. Philosophical Transactions of the Royal Society of London Series B: Biological Sciences. 2004;359(1452):1883–1893. doi:10.1098/rstb.2004.1557.

6. Nishikawa K. Titin: A Tunable Spring in Active Muscle. Physiology. 2020;35(3):209–217. doi:10.1152/physiol.00036.2019.

7. Gillespie DT. Exact Stochastic Simulation of Coupled Chemical Reactions. The Journal of Physical Chemistry. 1977;81(25):2340–2361. doi:10.1021/j100540a008.

8. Smith DA, Geeves MA, Sleep J, Mijailovich SM. Towards a Unified Theory of Muscle Contraction. I: Foundations. Annals of Biomedical Engineering. 2008;36(10):1624–1640. doi:10.1007/s10439-008-9536-6.

9. Pate E, Cooke R. A Model of Crossbridge Action: The Effects of ATP, ADP and Pi. Journal of Muscle Research and Cell Motility. 1989;10(3):181–196. doi:10.1007/BF01739809.

10. Dawson MJ, Gadian DG, Wilkie DR. Muscular Fatigue Investigated by Phosphorus Nuclear Magnetic Resonance. Nature. 1978;274(5674):861–866. doi:10.1038/274861a0.

11. Kushmerick MJ. Lessons for Muscle Energetics from 31 P NMR Spectroscopy. Myocardial and Skeletal Muscle Bioenergetics. 1986; p. 647–663.

12. Harris DE, Warshaw DM. Smooth and Skeletal Muscle Myosin Both Exhibit Low Duty Cycles at Zero Load in Vitro. Journal of Biological Chemistry. 1993;268(20):14764–14768. doi:10.1016/S0021-9258(18)82398-5.

13. O'Connell CB, Tyska MJ, Mooseker MS. Myosin at Work: Motor Adaptations for a Variety of Cellular Functions. Biochimica et Biophysica Acta (BBA) - Molecular Cell Research. 2007;1773(5):615–630. doi:10.1016/j.bbamcr.2006.06.012.

14. Bloemink MJ, Geeves MA. Shaking the Myosin Family Tree: Biochemical Kinetics Defines Four Types of Myosin Motor. Seminars in Cell & Developmental Biology. 2011;22(9):961–967. doi:10.1016/j.semcdb.2011.09.015.

15. Kee YS, Robinson DN. Motor Proteins: Myosin Mechanosensors. Current Biology. 2008;18(18):R860–R862. doi:10.1016/j.cub.2008.07.071.

16. Miller MS, Tanner BCW, Nyland LR, Vigoreaux JO. Comparative Biomechanics of Thick Filaments and Thin Filaments with Functional Consequences for Muscle

Contraction. Journal of Biomedicine and Biotechnology. 2010;2010:473423. doi:10.1155/2010/473423.

17. Millar NC, Geeves MA. Protein Fluorescence Changes Associated with ATP and Adenosine 5′-[γ-Thio]Triphosphate Binding to Skeletal Muscle Myosin Subfragment 1 and Actomyosin Subfragment 1. Biochemical Journal. 1988;249(3):735–743. doi:10.1042/bj2490735.

18. Bloemink MJ, Adamek N, Reggiani C, Geeves MA. Kinetic Analysis of the Slow Skeletal Myosin MHC-1 Isoform from Bovine Masseter Muscle. Journal of Molecular Biology. 2007;373(5):1184–1197. doi:10.1016/j.jmb.2007.08.050.

19. Ritchie MD, Geeves MA, Woodward SK, Manstein DJ. Kinetic Characterization of a Cytoplasmic Myosin Motor Domain Expressed in Dictyostelium Discoideum. Proceedings of the National Academy of Sciences. 1993;90(18):8619–8623. doi:10.1073/pnas.90.18.8619.

20. Nyitrai M, Rossi R, Adamek N, Pellegrino MA, Bottinelli R, Geeves MA. What Limits the Velocity of Fast-skeletal Muscle Contraction in Mammals? Journal of Molecular Biology. 2006;355(3):432–442. doi:10.1016/j.jmb.2005.10.063.

21. He ZH, Bottinelli R, Pellegrino MA, Ferenczi MA, Reggiani C. ATP Consumption and Efficiency of Human Single Muscle Fibers with Different Myosin Isoform Composition. Biophysical Journal. 2000;79(2):945–961. doi:10.1016/S0006-3495(00)76349-1.

22. Reggiani C, Potma EJ, Bottinelli R, Canepari M, Pellegrino MA, Stienen GJ. Chemo-Mechanical Energy Transduction in Relation to Myosin Isoform Composition in Skeletal Muscle Fibres of the Rat. The Journal of Physiology. 1997;502(Pt 2):449–460.

23. Kerrick WGL, Potter JD, Hoar PE. The Apparent Rate Constant for the Dissociation of Force Generating Myosin Crossbridges from Actin Decreases during Ca2+ Activation of Skinned Muscle Fibres. Journal of Muscle Research & Cell Motility. 1991;12(1):53–60. doi:10.1007/BF01781174.

24. Ferenczi MA, Homsher E, Trentham DR. The Kinetics of Magnesium Adenosine Triphosphate Cleavage in Skinned Muscle Fibres of the Rabbit. The Journal of Physiology. 1984;352(1):575–599. doi:10.1113/jphysiol.1984.sp015311.

25. Bárány M. ATPase Activity of Myosin Correlated with Speed of Muscle Shortening. Journal of General Physiology. 1967;50(6):197–218. doi:10.1085/jgp.50.6.197.

26. Sahlin K, Tonkonogi M, Söderlund K. Energy Supply and Muscle Fatigue in Humans. Acta Physiologica Scandinavica. 1998;162(3):261–266. doi:10.1046/j.1365-201X.1998.0298f.x.

27. Hoyle G. Muscles and Their Neural Control. John Wiley & Sons; 1983.

28. Bottinelli R, Reggiani C. Human Skeletal Muscle Fibres: Molecular and Functional Diversity. Progress in Biophysics and Molecular Biology. 2000;73(2):195–262. doi:10.1016/S0079-6107(00)00006-7.

29. Han YS, Geiger PC, Cody MJ, Macken RL, Sieck GC. ATP Consumption Rate per Cross Bridge Depends on Myosin Heavy Chain Isoform. Journal of Applied Physiology. 2003;94(6):2188–2196. doi:10.1152/japplphysiol.00618.2002.

30. Nag S, Trivedi DV. To Lie or Not to Lie: Super-relaxing with Myosins. eLife. 2021;10:e63703. doi:10.7554/eLife.63703.

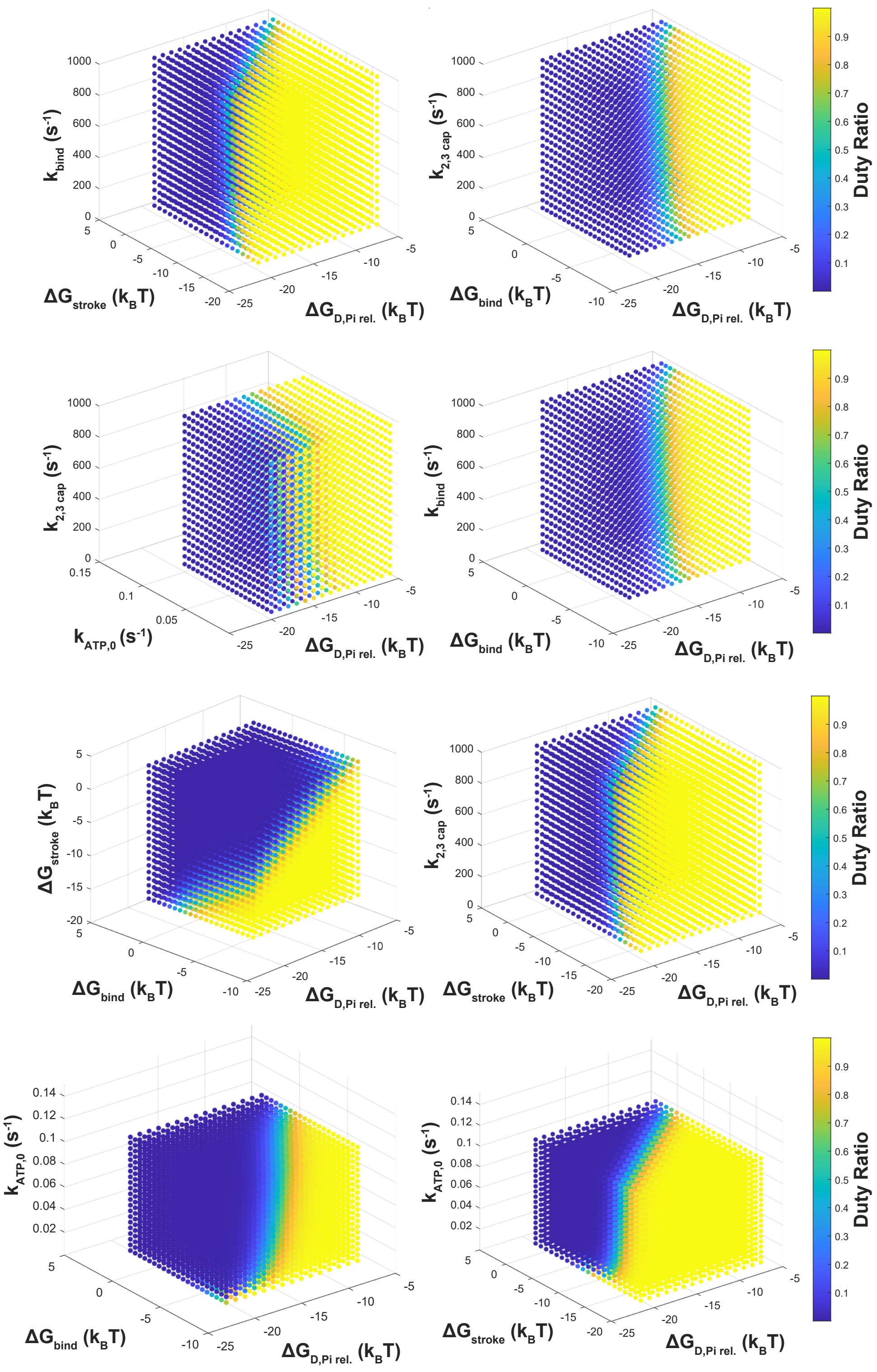

Fig B. (Part1) Parameter exploration for constants in rate equations. Each constant was evaluated within its relevant range against two other constants. The model output used to fit the parameters was the myosin duty ratio, which in skeletal muscle ranges from 3-5% based on previous studies.

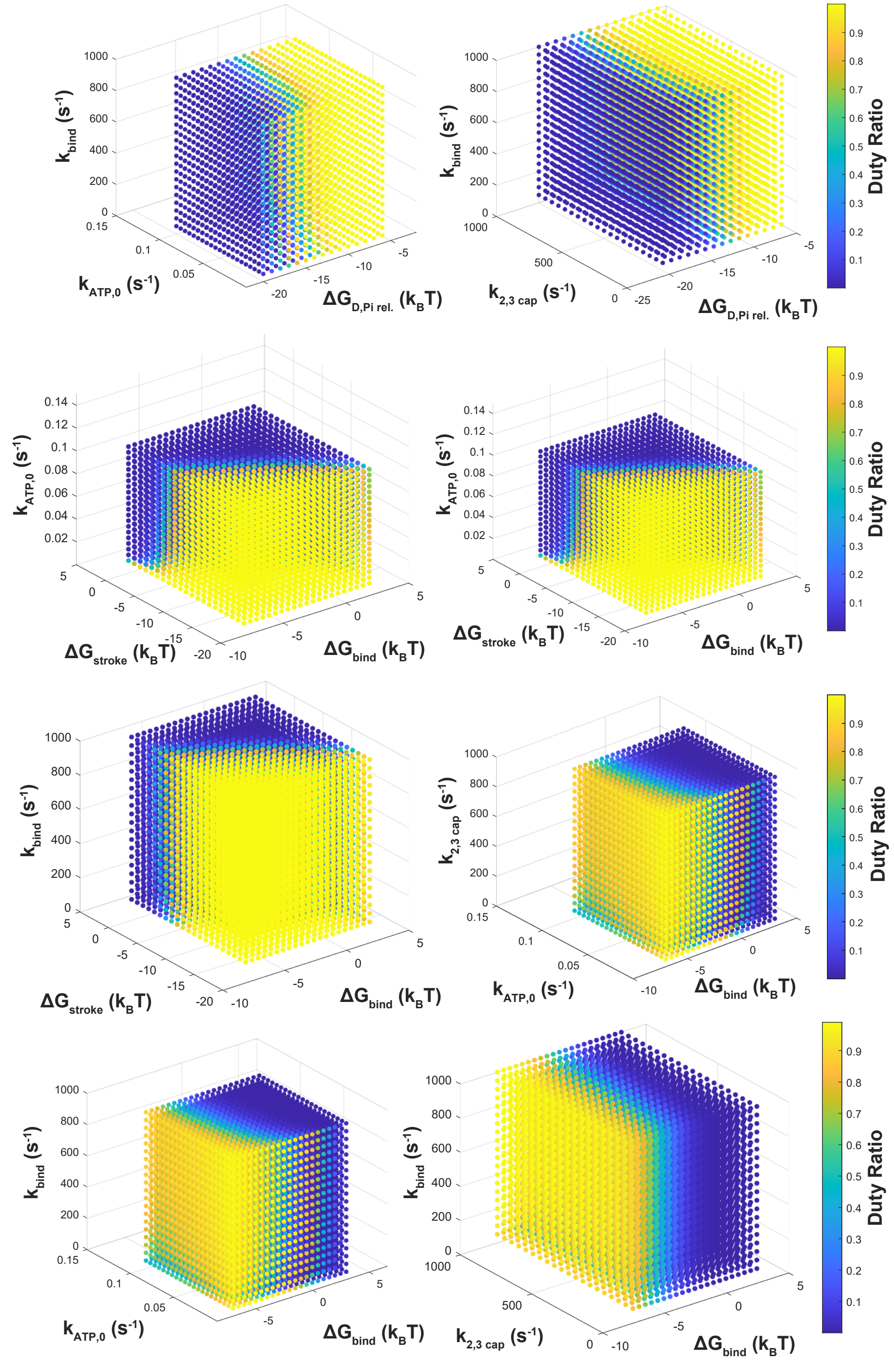

Fig C. (Part 2) Parameter exploration for constants in rate equations. Each constant was evaluated within its relevant range against two other constants. The model output used to fit the parameters was the myosin duty ratio, which in skeletal muscle ranges from 3-5% based on previous studies.

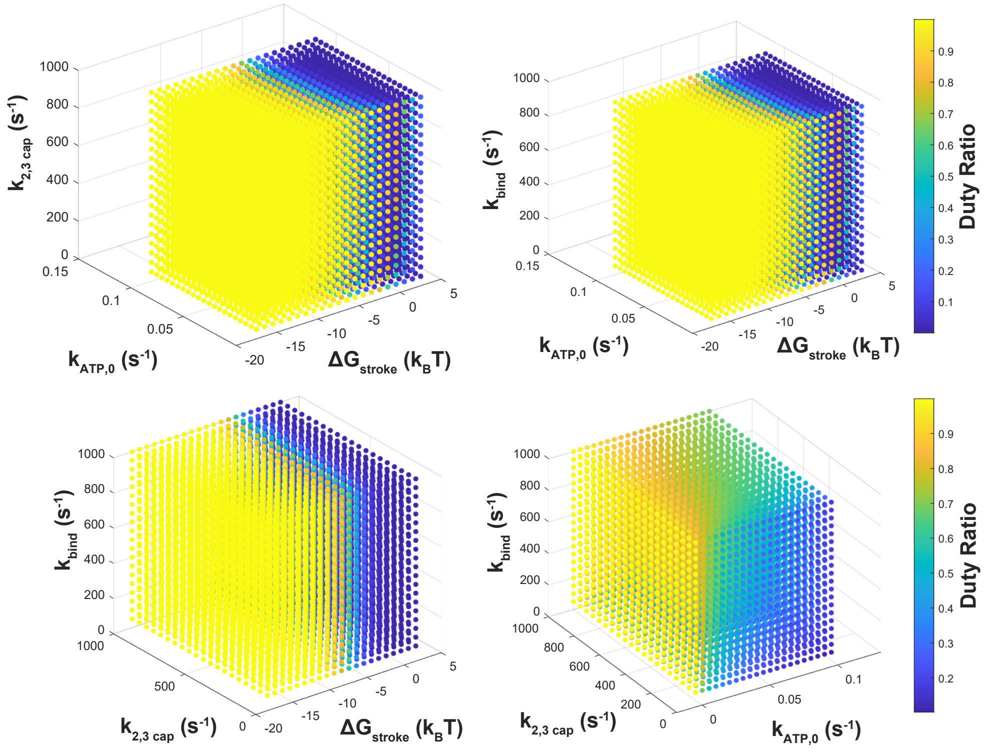

Fig D. (Part 3) Parameter exploration for constants in rate equations. Each constant was evaluated within its relevant range against two other constants. The model output used to fit the parameters was the myosin duty ratio, which in skeletal muscle ranges from 3-5% based on previous studies.
